# Mind captioning: Evolving descriptive text of mental content from human brain activity

**DOI:** 10.1126/sciadv.adw1464

**Published:** 2025-11-05

**Authors:** Tomoyasu Horikawa

**Affiliations:** Communication Science Laboratories, NTT, Inc., Kanagawa, Japan.

## Abstract

A central challenge in neuroscience is decoding brain activity to uncover mental content comprising multiple components and their interactions. Despite progress in decoding language-related information from human brain activity, generating comprehensive descriptions of complex mental content associated with structured visual semantics remains challenging. We present a method that generates descriptive text mirroring brain representations via semantic features computed by a deep language model. Constructing linear decoding models to translate brain activity induced by videos into semantic features of corresponding captions, we optimized candidate descriptions by aligning their features with brain-decoded features through word replacement and interpolation. This process yielded well-structured descriptions that accurately capture viewed content, even without relying on the canonical language network. The method also generalized to verbalize recalled content, functioning as an interpretive interface between mental representations and text and simultaneously demonstrating the potential for nonverbal thought–based brain-to-text communication, which could provide an alternative communication pathway for individuals with language expression difficulties, such as aphasia.

## Teaser

Nonverbal thoughts can be translated into verbal descriptions by aligning semantic representations between text and the brain.

## INTRODUCTION

Humans can recognize and recall intricate visual content comprising multiple semantic components, including objects, places, actions, and events, along with their interactions and relationships. These elaborate and structured mental representations form the foundation for translating thoughts into language and communicating experiences with others. Recently, substantial progress has been made in brain decoding of language-related information, enabling the direct production of linguistic outputs, such as text, from the human brain (*1*–*4*). However, decoding the perceptual—and not to mention mental—content associated with visual semantics to generate comprehensive descriptions of subjective experiences remains challenging. Translating brain activity linked to nonlinguistic semantic information, or thoughts, into verbal descriptions could greatly enhance our ability to interpret diverse mental states, opening up numerous possibilities for applications, particularly with text-prompt–based systems [e.g., ChatGPT (*5*) and Gemini (*6*)], as well as for scientific research.

Prior research on decoding visual semantics using human functional magnetic resonance imaging (fMRI) has focused on individual components or static images. This narrow focus has hindered the decoding of complex content involving interactions between multiple elements, thus obscuring our understanding of how the brain represents rich and structured visual semantics. While studies have successfully decoded individual components in viewed (*7*–*9*), imagined (*7*), and dreamed (*10*) content using object- or word-level features, they have fallen short of capturing neural representations of interactions and relationships that are not predicted by their individual components (*11*) and are crucial for recognizing actions and social interactions (*12*–*16*).

Some researchers have incorporated caption databases (*17*) or deep neural network (DNN)–based modules, such as nonlinear image captioning models (*18*–*20*), to produce sentence-level decoding predictions that appear to have linguistic structure. However, predictions based on database-search (DB-search) methods are limited to existing, often deliberately structured, descriptions that may not capture the full complexity of diverse visual content. In addition, nonlinear methods can introduce spurious information not “explicitly represented” in the brain (*21*–*24*). Specifically, nonlinear captioning models can construct sentence-like structured outputs even from object-level visual features (*25*), which inherently lack relational information. Thus, successful decoding via such models might reflect the complexities of the model architecture, rather than the properties of the underlying brain representations. Therefore, these approaches, designed to produce linguistically structured outputs, are not ideal for examining whether structured visual semantics, essential for representing relational information, are genuinely encoded in the brain through decoder outputs.

**Fig. 1.**
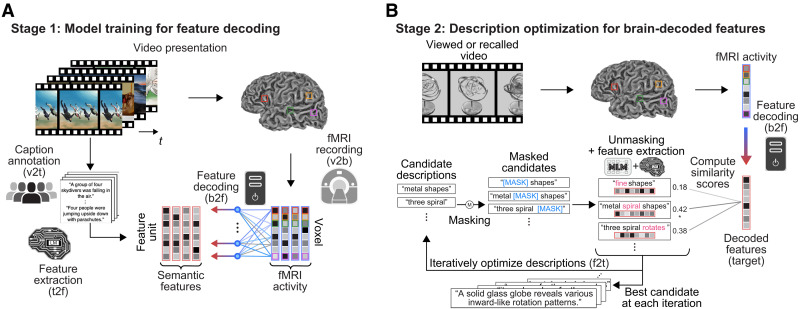
Mind captioning. Our method consisted of two stages. (**A**) We first trained linear decoding models to decode whole-brain fMRI activity, measured while each subject viewed videos, into semantic features from the captions of the videos using an LM (frozen). (**B**) We then used those models to decode brain activity induced by novel video stimuli or by recall-based mental imagery of those videos and optimized candidate descriptions iteratively by aligning their features with brain-decoded features through word replacement and interpolation, leveraging another LM pretrained for masked language modeling (MLM; frozen). The optimization consisted of three stages: masking, unmasking, and candidate selection. During masking, we randomly applied masks by replacing words with a mask or interpolating masks into the candidate word sequences. During unmasking, the MLM model created new candidates by filling in the masks in the masked candidates based on the context of surrounding words. During candidate selection, we computed semantic features of all new and original candidates using the LM for feature extraction. We then evaluated the similarity between those candidate features and the target brain-decoded features to select the top candidates for further optimization. The optimization process was initiated from a noninformative word (“<unk>”) to avoid incorporating any prior assumptions for description generation and was repeated 100 times. See fig. S2 for details on the model and parameter validations. Each transformation process (e.g., v2t and b2f) corresponds to a transformation ID summarized in Table 1. v2t, video to text; t2f, text to feature; v2b, video to brain; b2f, brain to feature; f2t, feature to text. Because of copyright restrictions, the actual video frames used in the experiments have been replaced with schematic illustrations throughout this paper. See Materials and Methods for details of how these illustrations were generated.

To overcome these limitations, we introduce a generative decoding method called “mind captioning,” which generates descriptive text mirroring semantic information represented in the brain (Fig. 1 and Table 1). Our method combines linear feature decoding analysis (*7*, *9*, *10*), using semantic features computed by a deep language model (LM), with a novel optimization method that generates text based on these features. Semantic features serve as intermediate representations for decoding (translating) semantic information from the brain into text. They can act as a bridge for decoding both perceptual and mental content, as shared representations exist between visual perception and mental imagery, particularly for high-level information (*7*, *10*, *26*–*28*). In addition, deep LMs offer the advantage of effectively capturing contextual meanings, which are crucial for delineating intricate interrelationships (*29*–*34*).

**Table 1.** Summary of information modalities and their transformations. Transformation IDs [e.g., v2t (video to text), b2f (brain to features), etc.] are used consistently in Fig. 1 to denote each transformation process.

Transformation ID	Input modality	Output modality	Transformation method
v2t (video to text)	Videos	Text captions	Crowdsourced manual annotation of text captions
t2f (text to feature)	Text captions	Semantic features	Feature extraction using a pretrained LM (e.g., DeBERTa-large)
v2b (video to brain)	Videos	Brain activity	fMRI recording
b2f (brain to feature)	Brain activity	Semantic features	L2-regularized linear regression
f2t (feature to text)	Semantic features	Text captions	Iterative text optimization using an MLM model (e.g., RoBERTa-large)

The challenge lies in linguistically interpreting the information in semantic features decoded from brain activity. Although the ideal approach would involve examining all possible word sequences to identify the description whose semantic features best match the decoded features, this is not feasible because the number of candidates is infinite. We thus developed an iterative optimization method that generates descriptive text from scratch by progressively aligning the semantic features of candidate descriptions with target brain-decoded features through word replacement and interpolation in a search for the best description (Fig. 1B). Crucially, we leveraged an LM pretrained for masked language modeling (MLM) (*35*) to constrain the search space during optimization. By directly optimizing word sequences to match brain-decoded features, our method minimizes dependence on external resources such as caption databases or nonlinear captioning models, thereby ensuring the generation of descriptions more closely aligned with brain representations while maintaining the interpretability of structured visual semantics in the brain.

To demonstrate the effectiveness of our method, we first validated it for perceptual content by constructing decoding models (decoders) from stimulus-induced brain activity and then tested their generalizability to activity during recall-based mental imagery. Specifically, we measured brain activity in six subjects—all Japanese individuals who were non-native English speakers—using fMRI, while they viewed or recalled video clips (fig. S1) (*36*) and created a data sample by averaging fMRI volumes measured during viewing and recalling each video. To enhance the quality of the fMRI data, we averaged data samples over five repetitions for each stimulus or imagery item in the test phase. Using the samples from stimulus-induced brain activity, we trained decoders to predict semantic features, which were computed from corresponding video captions using an LM [DeBERTa-large (*37*)]. We then used these decoders to translate brain activity associated with viewed and recalled content into semantic features for novel test videos not used during training. Last, we used the decoded features to optimize the text using an MLM model [RoBERTa-large (*38*)]. Through these analyses, we aimed to validate our method’s capability to generate comprehensive descriptions of both viewed and recalled content from brain activity, establishing a framework for decoding nonverbal mental content and exploring the neural basis of structured visual semantics.

## RESULTS

### Generating viewed content descriptions

**Fig. 2.**
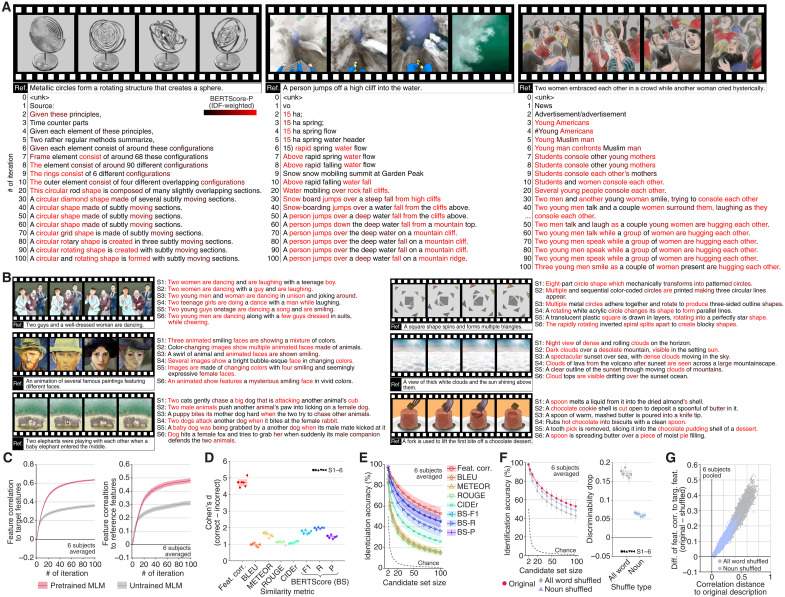
Generating viewed content descriptions. Descriptions were generated using features from all LM layers decoded from whole-brain activity. (**A**) Evolved descriptions during the optimization (see https://horikawa-t.github.io/MindCaptioningProject/ for more results with the original videos). (**B**) Descriptions after 100 iterations for all subjects (see fig. S3A for more example). In (A) and (B), the color indicates accuracy [inverse document frequency (IDF)–weighted BERTScore-P]. A reference caption of the video is shown below frames. (**C**) Feature correlations between features of generated descriptions and those decoded from the brain, as well as those computed from correct references. (**D**) Cohen’s *d* of discriminability (see fig. S4B for raw scores). Feat. corr., Feature correlation. (**E**) Video identification accuracy with varying numbers of candidates. (**F**) Effects of word-order shuffling on video identification accuracy and discriminability. (**G**) Scatterplot of the correlation distances (one minus feature correlation) between the original and shuffled descriptions against the difference in feature correlations to target features between original and shuffled descriptions. Each dot indicates a shuffled description. Shades in (C) and (E) and error bars in (D) and (F) indicate 95% confidence intervals (CIs) across samples (*n* = 72). Shades in (D) and (F) indicate 95% CI across subjects (*n* = 6). See fig. S4 for individual results.

The optimization of text, based on decoded features from stimulus-induced brain activity, resulted in a progressive evolution of descriptive texts (Fig. 2A). Initially, the descriptions were fragmented and lacked clear meaning. However, through iterative optimization, these descriptions naturally evolved to have a coherent structure and effectively capture the key aspects of the viewed videos. Notably, the resultant descriptions accurately reflected the content, including the dynamic changes in the viewed events (Fig. 2B). Furthermore, even when specific objects were not correctly identified, the descriptions still successfully conveyed the presence of interactions among multiple objects (e.g., Fig. 2B, bottom left).

Throughout the optimization process, the features of the evolved descriptions exhibited increasingly stronger correlations with the target features and, consequently, with the features of the reference captions annotated on the viewed videos (Fig. 2C). In contrast, the same optimization performed with an untrained MLM model, which randomly suggests candidate words during unmasking—a process analogous to a genetic algorithm—did not yield comparable results (fig. S3B). These results emphasize the significance of contextual information from a pretrained MLM model to efficiently explore descriptions closely aligned with brain representations and to enhance description quality.

To assess decoding performance, we used multiple similarity metrics to evaluate the similarity between the generated descriptions and the reference captions. We calculated similarity scores for both the captions of the viewed (correct) and irrelevant (incorrect) videos and defined discriminability as the difference between them. The generated descriptions exhibited significantly high discriminability across all metrics and subjects [Fig. 2D; Wilcoxon signed-rank test, one-tailed, *P* < 0.01, false discovery rate (FDR) corrected across metrics and subjects], indicating that these descriptions were accurate enough to differentiate video content.

To gain an intuitive understanding of the performance, we evaluated the accuracy of video identification by comparing the generated descriptions to both correct and incorrect captions across various numbers of candidates. Performance consistently exceeded chance levels for all set sizes, with ~50% accuracy among 100 distinct possibilities for all subjects when using feature correlation (chance = 1%; Fig. 2E), demonstrating the effectiveness of our method in translating detailed information from the brain into text through semantic features.

To further highlight the effectiveness of our method, it is worth noting that it surpasses existing approaches and holds promise for continued improvement. Specifically, our method captured subjective experiences more accurately and flexibly (fig. S5), as its generated descriptions aligned more closely with captions that individual subjects rated as highly consistent with their perceptions (figs. S1A and S5E). In addition, our method demonstrated superior discriminability compared to approaches relying on caption databases (*17*) or nonlinear captioning models (*18*) across both video- and image-induced fMRI data (*39*) (figs. S6 and S7). Moreover, our method robustly generated descriptions that accurately reflected the viewed content regardless of the LMs used, and brain encoding performance—a metric assessing alignment between the brain and models (*31*, *40*)—correlated with the text generation performance of the LMs (fig. S8). These results suggest that using LMs more closely aligned with the brain [e.g., GPT3 (*5*), OPT (*41*), or LLaMA (*42*)] may further improve the effectiveness of our method.

### Assessing structured semantic information in generated descriptions

A key advantage of structured descriptions over simple word lists is their ability to organize words to convey contextual meaning that goes beyond a mere list of individual entities, capturing their interrelations. To capture analogous structures in visual experiences, we define structured visual semantics as a range of visually grounded relational structures—including semantic roles, attribute-object relations, and spatial configurations—illustrated by contrasts such as “a bird eats a snake” versus “a snake eats a bird” or “some grass in a mug” versus “a mug in some grass,” which are linguistically describable and sensitive to word order (*43*). These distinctions are essential for building rich representations of the visual structure—such as action direction and visually implied interrelations—that form the foundation for perceiving social and human-object interactions (*11*–*16*).

To assess whether our generated descriptions captured the relational structure of visual scenes through proper word arrangement, we tested the effect of shuffling their word order—either for all words or nouns only (up to 1000 shuffled variants; see Materials and Methods)—under the hypothesis that if the original word order accurately conveyed visual relationships, then shuffling would reduce discriminability.

While the shuffled descriptions retained reasonably high accuracy in identifying videos—indicating that word lists alone provide informative cues—shuffling all words, or even just the nouns, significantly impaired discriminability (Fig. 2F; Wilcoxon signed-rank test, one-tailed, *P* < 0.01, FDR corrected across subjects). This reduction in discriminability remained robust even when using the minimally disrupted shuffled descriptions, which had the highest fluency (or linguistic acceptability) as assessed by the pseudo–log-likelihood score from MLM scoring (*44*, *45*) (fig. S4F). These results demonstrate that our method generates descriptions that capture more detailed information than simple word lists.

Notably, the impact of shuffling was more pronounced when using features from deeper LM layers to generate descriptions (fig. S9), underscoring the importance of the deep structure of the LMs in constructing contextual semantic representations and accurately decoding structured information.

### Disentangling the origins of descriptive coherence

A crucial step in understanding the representational basis of our method is to determine whether the coherence of generated descriptions—essential for depicting visual relations—reflects structured semantic information in the target features or is instead artificially imposed by the priors from the MLM model used in the text optimization process (Fig. 1B). We reasoned that if brain-decoded features do genuinely represent specific structured semantic information—uniquely conveyed by the particular word order of the generated description—then these features should exhibit greater similarity to the features of the original description than to those of shuffled variants that preserve the same words but disrupt their original order. We tested this by comparing feature correlations between the brain-decoded features and both the original and shuffled descriptions.

Supporting our reasoning, shuffling the word order markedly reduced feature correlations, especially when it substantially altered the original meanings (Fig. 2G). The original descriptions scored highest among all variants generated through all-word shuffling and ranked in the top 0.001% for noun-only shuffled variants (six subjects pooled). These results suggest that the specific word order of the generated descriptions is meaningfully guided by the semantic information encoded in the feature representations.

To further explore the possibility that the MLM model imposed coherent structure on the generated descriptions, we examined the outputs of decoders trained with semantic features from word-order–shuffled captions—a method intended to prevent the decoders from learning structured semantics. As a result, we confirmed that incoherent word sequences were generated, although they still contained words that semantically matched individual components of the viewed videos (fig. S3C).

We also confirmed that our text optimization method accurately reconstructs original descriptions using model-derived features computed from the reference captions (i.e., not brain-decoded features) after sufficient iterations (fig. S10). Reconstructions that matched the target captions yielded feature correlations of 1.0, whereas even minor deviations—such as word omissions or reordering—led to notable reductions in correlation, highlighting the fidelity of the semantic feature space. Although the method struggled with long or shuffled captions—likely due to the incremental, token-based optimization strategy and residual priors in the MLM model—these priors alone were insufficient to enforce coherent word sequences. These results indicate that the semantic feature space preserves fine-grained information sufficient for accurate text reconstruction, with the model priors playing a supportive—but not determinative—role in shaping the linguistic form of the output.

Together, these results reinforce our finding that the text optimization process does not impose restrictive constraints on forming linguistically structured outputs when generating coherent and structured descriptions from brain activity. Instead, the observed coherence is likely guided by the structured information present in the brain-decoded features, indicating that these features—not the optimization algorithm—are the primary source of the generated structure.

### Contributions from different brain areas

Having confirmed the ability to generate accurate and well-structured descriptions from whole-brain activity, we next examined the contributions of specific brain regions to this decoding, focusing on whether complex, structured semantics can be derived independently of the language network. Although numerous studies have shown that a broadly distributed semantic network encodes the meanings of both visual and linguistic information (*46*–*49*), most have focused on category- or word-level representations. Research on structured semantics has primarily concentrated on language processing, often associated with the frontotemporal language network (*34**,* *50**,* *51*), whereas the neural substrates for structured visual semantics remain comparatively underexplored. Emerging evidence suggests that, while the language network is also recruited in processing meaningful visual scenes (*52**,* *53*), other regions—particularly the lateral occipital temporal cortex—encode certain relational aspects of visual information, including interactions and the directedness of actions among persons and objects (*11*–*16*). These findings suggest the intriguing potential to enable the direct decoding and communication of rich, structured semantic information from brain activity while bypassing the linguistic processing typically required to translate thoughts into words. Pursuing this possibility could broaden the scope of brain-machine interfaces (BMIs) for converting nonverbal thoughts into text, opening avenues for semantic decoding that do not rely on language.

**Fig. 3.**
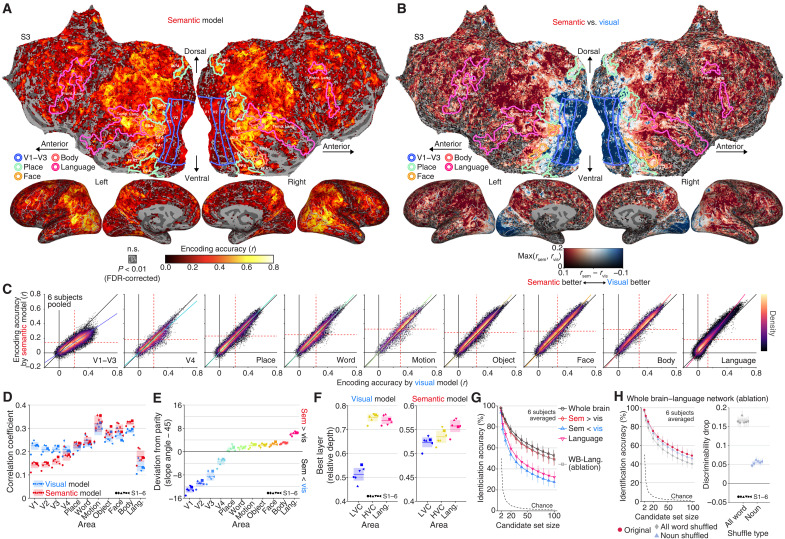
Contributions from different brain areas. A cross-validation analysis was performed within training perception data. Encoding models were trained using features from each layer to generate predictions from multiple layers. The final predictions were constructed on the basis of the best layer per voxel determined using nested cross-validation. (**A**) Encoding performance of the semantic model. n.s., not significant; Front. Lang., Frontal language area; Temp. Lang., Temporal language area. **(B**) Performance difference between the semantic and visual models. sem, semantic; vis, visual. (**C**) Density heatmap of accuracy (solid lines; best linear fit). (**D**) Mean encoding accuracy within each area. (**E**) Deviation from parity based on the slope angles of the best linear fit. (**F**) Mean of the best layers. The indices of layers with the highest performance were averaged across voxels in each area and then converted into relative depth. (**G**) Video identification accuracy from different brain areas. Decoders were trained using voxels with higher encoding accuracy according to the semantic or visual models, voxels within the language network, or voxels from the entire brain except for the language network. WB, whole brain. (**H**) Effects of word-order shuffling on video identification accuracy and discriminability without using the language network. In (D) to (H), error bars and shades indicate 95% CI across samples and subjects, respectively. See figs. S4 and S11 for individual results.

To establish a foundation for evaluating decoding performance, we began by analyzing how the brain encodes structured visual semantic information in videos. We constructed two encoding models: one based on the semantic features used in our decoding analysis and the other on visual features from a video recognition DNN pretrained to classify object and action categories [TimeSformer (*54*)]. The semantic encoding model effectively predicted brain activity in the language network and in regions involved in recognizing objects, actions, and interactions (*11*–*16*), spanning the higher visual cortex (HVC) and extended areas of the parietal and frontal cortices (Fig. 3A). While the visual model performed better in the lower visual cortex (LVC; V1 to V3), the semantic model progressively outperformed it in the HVC and language network (Fig. 3, B to E). The shift in relative superiority between the visual and semantic models occurs at the midpoint of the category-selective regions, between their posterior and anterior halves, suggesting a functional boundary (Fig. 3B). In addition, voxels in the HVC and language network were better predicted by features from deeper layers of the visual DNN and the LM (Fig. 3F). Notably, the LM layers yielding the highest performance for language network voxels were much deeper than those for the HVC (Wilcoxon rank sum test, one-tailed, *P* < 0.01, FDR corrected across subjects), highlighting an indicative link between the language network and contextualized semantic information. These results demonstrate that the language network, along with other regions, is involved in encoding contextual semantics, consistent with previous studies on its activation by nonverbal visual semantics (*52**,* *53*).

We then assessed decoding performance using video identification analysis based on descriptions generated from these brain regions with varying selectivity (Fig. 3G). Focusing on voxels better predicted by the semantic model yielded higher performance, approaching whole-brain activity results for some subjects (fig. S4H). In contrast, decoding from voxels better predicted by the visual model resulted in weaker performance, indicating limited contributions from these voxels, although they are widely distributed across the posterior side of visual category-selective areas. Using only the language network, despite its involvement in encoding contextual semantics, did not produce high performance, suggesting that its contribution may be more supportive than essential. Notably, ablating the language network did not profoundly affect performance, achieving almost 50% accuracy from 100 possibilities (chance = 1%; six subjects averaged), showing decreased accuracy and discriminability by word-order shuffling (Fig. 3H; Wilcoxon signed-rank test, one-tailed, *P* < 0.01, FDR corrected across subjects), and even generating intelligible descriptions (fig. S3D).

These results suggest that accurate descriptions capturing structured semantics can be generated without relying on the language network, indicating that structured visual semantic information is represented across regions extending from the anterior portions of the occipital visual cortex into parietal and frontal cortices outside the language network. These representations may underlie the comprehension of complex visual events in individuals with global aphasia (*52*). These findings also provide further support for the distinction between language and nonverbal thought (*55*).

### Generating recalled content descriptions

**Fig. 4.**
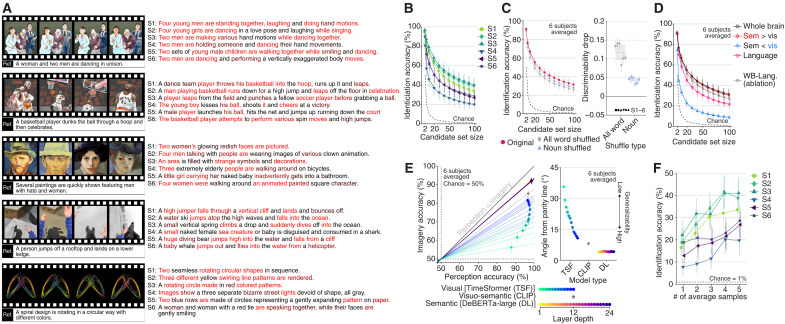
Generating recalled content descriptions. We applied decoders trained on stimulus-induced brain activity (DeBERTa-large; all layers; decoded from whole-brain activity, unless otherwise stated) to analyze the brain activity of subjects engaged in recalling video scenes from memory, prompted by verbal descriptions (see fig. S1B). (**A**) Descriptions of recalled content generated after 100 iterations for all subjects (see fig. S3E for more examples). (**B**) Identification accuracy of recalled videos for individual subjects. (**C**) Effects of word-order shuffling on identification accuracy and discriminability of recalled videos. (**D**) Identification accuracy of recalled videos from different brain areas. (**E**) Comparison of feature-based pairwise video identification accuracy between perception and imagery. Generalizability was quantified as the angle between the parity line (the diagonal line indicating equal accuracy for perception and imagery) and a line connecting the chance level (50%) with the observed accuracy for each model and layer. Error bars (left and right panels) indicate 95% CI across samples and jackknife-estimated standard errors, respectively. (**F**) Identification accuracy of recalled videos with a varying number of averaged samples. For (A), (C), and (D), conventions are the same as for Figs. 2 (B and F) and 3G. See figs. S6 (B and D) and S12 to S14 for additional results.

Last, we investigated whether the decoders trained on brain activity induced by visual stimuli—hereafter referred to as perception-trained decoders—could be used to generate descriptions of mental content based on brain activity induced by mental imagery of recalled videos, applying the same evaluation procedures as in the perception data analysis. The analysis successfully generated descriptions that accurately reflected the content of the recalled videos, although accuracy varied among individuals (Fig. 4A). These descriptions were more similar to the captions of the recalled videos than to irrelevant ones (fig. S12, A and B), with proficient subjects achieving nearly 40% accuracy in identifying recalled videos from 100 candidates (Fig. 4B; chance = 1%). Shuffling word order resulted in a notable reduction in video identification accuracy and discriminability (Fig. 4C; Wilcoxon signed-rank test, one-tailed, *P* < 0.01, FDR corrected across subjects). Excluding the language network from the analysis slightly, but not substantially, reduced video identification accuracy (Fig. 4D).

We further confirmed that semantic features serve as effective intermediate representations, allowing perception-trained decoders to generalize across cognitive states and decode mental content (Fig. 4E). Specifically, we applied perception-trained decoders to both stimulus- and imagery-induced brain activity, evaluating feature-based pairwise video identification performance using features from individual layers of three model types—visual (TimeSformer) (*54*), visuo-semantic (CLIP) (*56*), and semantic (DeBERTa-large) (*37*). When quantifying generalizability as the angle between the parity line and a line connecting chance level (50%) with the observed accuracies, generalizability increased with layer depth in visual features, was relatively high for visuo-semantic features, and was highest for semantic features across all layers. These results demonstrate that semantic features robustly bridge neural representations shared across perception and imagery, providing a reliable foundation for translating internal experiences into coherent text.

Notably, when the same text generation analysis was applied to brain activity during the preparation period (fig. S1B), all subjects showed lower accuracy than during the imagery period, with most subjects (except S2) performing at levels closer to chance (fig. S12D). This finding suggests that volitional mental imagery, rather than text reading of verbal cues, is essential for eliciting semantic neural representations, enabling the accurate generation of target content descriptions.

Collectively, these results confirm the generalizability of the perception-trained decoders to generate descriptions of recalled content, showcasing their capability to effectively verbalize mental representations. In addition, we were able to obtain comprehensible descriptions of the recalled content with reasonable identification accuracy from single-trial fMRI activity (Fig. 4F and fig. S14), demonstrating the potential applicability of our method to experiences that are difficult to reproduce, such as dreams (*27*).

## DISCUSSION

We successfully generated descriptive text representing visual content experienced during perception and mental imagery by aligning semantic features of text with those linearly decoded from human brain activity. Our success is attributed to two key factors: the advancement of deep LMs that provide contextualized semantic representations similar to those in the human brain (*29*–*34*) and our innovative text optimization method using the MLM model for word-level optimization while efficiently constraining the search space. Together, these factors facilitate the direct translation of brain representations into text, resulting in optimally aligned descriptions of visual semantic information decoded from the brain. These descriptions were well structured, accurately capturing individual components and their interrelations without using the language network, thus suggesting the existence of fine-grained semantic information outside this network. Our method enables the intelligible interpretation of internal thoughts, demonstrating the feasibility of nonverbal thought–based brain-to-text communication.

Our methodological configuration was designed to generate accurate and comprehensive descriptions of mental content from brain activity while facilitating interpretation of the neural basis of structured visual semantics. Rather than mapping brain activity directly to text (*19*, *20*)—a strategy requiring large-scale brain data and introducing architectural complexity that complicates attribution—we adopted a two-stage approach: first, decoding brain activity into a feature space, and then generating text from these features, thereby enabling localization of potential information loss. We used stimulus-induced activity to ensure stable decoder training and used linear mappings to probe explicit brain representations (*21*–*24*), prioritizing interpretability and mitigating overfitting—particularly crucial for generalizing to imagery. We aligned brain activity with semantic features to leverage high-level representations shared between perception and imagery (*7*, *10*, *26*–*28*), enhancing cross-state generalizability. Indeed, semantic features demonstrated superior generalizability when decoding imagery using perception-trained decoders, outperforming visual and visuo-semantic counterparts—commonly used in captioning models (*25*, *57*) and their decoding applications (Fig. 4E) (*18*). We used an iterative process to generate descriptions that accurately reconstructed original descriptions from model-derived features—thereby supporting both the fidelity of the semantic feature space and the efficacy of the optimization (fig. S10). The method generates descriptions optimally aligned with brain-decoded features, eliminating reliance on caption databases (*17*) or learned feature-to-text mapping modules (*18*)—both dependent on curated, labeled datasets that limit output diversity and blur attribution to brain-derived features—and consequently outperformed these alternatives across both video and image datasets (figs. S5 to S7). Together, our approach balances interpretability, generalizability, and performance—establishing a transparent framework for decoding nonverbal thought into language and paving the way for systematic investigation of how structured semantics are encoded across the human brain.

A key feature distinguishing our method from previous visual-semantic decoding approaches is its generative capability—producing descriptions semantically aligned with brain-decoded features without relying on external resources (*17*, *18*)—stemming from our iterative optimization-based generation process. Unlike one-directional, single-pass generation with autoregressive LMs (*3*, *4*), our use of a bidirectional MLM model (*35*) enables full contextual integration from both directions and mitigates model-prior bias through iterative feature-guided optimization—achieving high-fidelity verbalization whenever the target representation lies within the searched space (fig. S10). Notably, this optimization process is robust enough to generate descriptions even from a noninformative initial state (i.e., <unk> token; Fig. 2). While some limitations remain, emerging diffusion-based generators (*58**–**60*) may further expand the search space and enhance descriptive fidelity. Requiring curated labels only at the decoder-training stage—a scalable design with minimal supervision—our framework is readily extensible beyond the visual domain to other sensory modalities (e.g., audition and touch) and cognitive domains (e.g., numerical reasoning and conceptual thought), offering a versatile framework for semantically grounded brain decoding across diverse forms of mental content.

While our method generates linguistic outputs through brain decoding, it differs from previous language decoding attempts, as it does not rely on brain activity associated with language production (*2*, *4*) and perception (*1*, *3*). Instead, we trained decoders on brain activity induced by nonlinguistic visual stimuli to predict semantic features linked to the visual content of viewed videos. This approach enabled the generation of descriptions for both viewed and recalled content without involving the language network (Figs. 3 and 4). Notably, all subjects in our study were non-native English speakers—specifically, native Japanese speakers—nevertheless, our method proficiently generated text outputs in English. These results illustrate that our method can directly translate nonlinguistic brain semantics into linguistic descriptions, even when the subject is neither native nor proficient in the output language. Consequently, it can be applied to decode brain activity in nonlinguistic subjects, including infants and animals, providing insights into how they develop the neural basis for processing complex visual semantics.

Moreover, by enabling the translation of nonverbal visual semantics in the brain into text, our method opens new communication channels in BMI applications, extending possibilities beyond traditional approaches for individuals with language or motor impairments. For instance, this approach could serve as an effective means of communication for individuals with aphasia, who struggle with language expression due to damage in language areas. Furthermore, our method complements visual-based BMI systems (*61*) by providing an alternative communication pathway for individuals with conditions such as amyotrophic lateral sclerosis, where degeneration of motor-related activity limits the effectiveness of motor-based BMIs. Thus, our method holds the potential to enhance communication and interaction in clinical and assistive settings.

The present study sheds light on the neural bases of structured visual semantics by examining the effects of ablating the language network and the posterior parts of category-selective regions during text generation analysis (Fig. 3). Excluding these regions did not substantially affect the quality of the generated descriptions, despite the broad distributions of semantic-feature–predictive voxels across the cortex, including within these regions—consistent with previous studies (*46*–*49*). This outcome suggests that brain areas outside these regions may contribute to representing structured visual semantics and aligns with research on visual representations involved in recognizing interactions and actions (*11*–*16*). Our findings build on these studies by demonstrating that such representations are sufficient for constructing fine-grained, cohesive descriptions of both viewed and recalled content.

Furthermore, our encoding analysis, which contrasts models based on LM-derived contextual semantic features with visual DNN features related to object and action categories, revealed a functional boundary within the category-selective areas that separates posterior from anterior regions (Fig. 3B). Although this analysis specifically focused on neural representations linked to visual stimulus perception, this boundary coincides with known distinctions between visual and linguistic semantics (videos versus audio stories) (*62*) and between perceptual and mnemonic systems (*63*). Consequently, our findings suggest an alternative perspective on this boundary: Posterior regions may primarily support isolated semantic (or categorical) representations, while anterior regions integrate these into contextualized representations. The proximity of these anterior regions to language areas suggests that they may play a bridging role, transforming nonverbal information into verbal expressions and connecting nonverbal and verbal semantics. Further research is needed to clarify how these anterior regions interact with the language network to achieve this integration.

While our method has shown the ability to generate descriptions that resemble captions rated by the subjects as highly consistent with their perceptions (fig. S5E), there remains potential for improvement in capturing the full spectrum of subjective experiences, particularly by refining the alignment and depth of captions annotated to video stimuli. Specifically, we relied on captions provided by independent annotators, which may not fully align with each subject’s unique perceptions, potentially affecting decoding performance. Although our use of rich annotations for each video (20 captions per video) likely mitigated some variability, training decoders on subjects’ own reports might yield even closer alignment. Furthermore, because we instructed annotators to focus on visual content rather than subjective aspects such as emotional reactions (*64*), the generated descriptions were predominantly concrete and rarely reflected abstract dimensions such as impressions and emotions (*65*). With annotations that more accurately reflect and encompass various dimensions of subjective experience, our method may capture the content of a subject’s mind more comprehensively.

A limitation of our study is the use of natural videos sourced from the web (*36*). While this approach enhances ecological validity, it constrains our ability to precisely identify the relational structures captured by our method and to assess its generalizability to atypical scenes (e.g., “a man bites a dog”). Notably, the word orders of the generated descriptions were effectively optimized to align with brain-decoded features (Fig. 2G), yielding descriptions with higher discriminability than their shuffled variants (Fig. 2F). Although these results suggest that the generated descriptions reflect some relational structure, the lack of experimental control makes it difficult to determine which relational structures are being captured. Critically, it remains unclear whether the success of our method reflects true generalizability beyond common relational patterns or instead relies on implicit biases toward typical scene structures. This potential bias could be introduced at any stage of the pipeline, such as through model priors, training data distribution, or the experimental design—including stimulus selection. Future studies should address these issues by incorporating strong out-of-distribution probes using controlled stimuli that systematically manipulate distinct relational structures—including contrasting or atypical configurations (*43**,* *66*). We consider such experiments a critical next step for a more rigorous assessment of both the type of relational information decodable from brain activity and the generalization capacity of our method across a broader range of relational contexts.

A potential concern with our demonstration of generating mental content descriptions is that the verbal prompts used to cue the target videos may have influenced brain activity during the imagery period due to the slow hemodynamic response. During the preparation period, as subjects might have started to recall the videos while reading these prompts, it is difficult to fully differentiate brain activity associated with text reading from that related to mental imagery. However, because our decoders were specifically trained on brain activity induced by nonlinguistic visual stimuli, they prioritize semantic information directly linked to visual content over linguistic cues. Furthermore, our analysis showed that descriptions generated during the imagery period were of higher quality than those from the preparation period (fig. S12, C and D), suggesting that volitional mental imagery effectively recruited the neural representations necessary for accurate descriptions. Nonetheless, future investigations applying our method to spontaneous mental imagery (e.g., mind wandering or dreaming) using subjects’ verbal reports as a reference would be necessary to clarify its ability to generate descriptions of mental content free from the influence of external stimuli.

To accurately characterize our primary contribution, it is essential to frame our method as an interpretive interface rather than a literal reconstruction of mental content. While our approach enables the linguistic interpretation of nonverbal mental representations, the resulting outputs inevitably reflect not only brain-derived information but also prior knowledge of the world—often implicitly embedded in the experimental and modeling framework. These framework-dependent priors manifest in several aspects discussed above—such as the choice of LMs, the language and type of annotations (e.g., English versus Japanese and visual versus emotional), and the properties of experimental materials—all of which collectively shape how brain representations are ultimately verbalized, particularly under supervised decoder training. For example, our finding that decoders trained on coherent and incoherent captions yielded correspondingly structured or unstructured outputs (Fig. 2 and fig. S3C) illustrates how the generated text is shaped by the world knowledge provided during training. Thus, the decoded content should be viewed not as a pure readout or reconstruction of brain states but as a translation filtered through the lens of a specific interpretive framework. Our key contribution, therefore, should be understood not as the faithful recovery of the brain’s intrinsic “language” but as the construction of a versatile and expressive pathway for interpreting nonlinguistic mental representations—by leveraging the universality of natural language as instantiated through the semantic expressiveness and generative power of LMs. This interpretive bridge advances the field by enabling more flexible and nuanced renderings than previously possible while simultaneously raising fundamental questions: How much of the decoded output truly originates in the brain, and how much reflects the constraints of our tools? Would decoding fidelity improve with more brain-aligned semantic spaces? How might an individual’s linguistic profile—their native language, vocabulary, syntax, and expressive habits—systematically shape the decoded output? Addressing these questions requires not only improving decoding performance but also critically examining the epistemological assumptions inherent in the frameworks we use to interpret the mind.

The generation of mental content descriptions was successful even from single-trial fMRI activity of mental imagery (Fig. 4F and fig. S14). However, this success raises ethical concerns regarding potential invasions of privacy. Key issues include the risk of unintentionally disclosing primitive thoughts before individuals have chosen to verbalize them. In addition, unwanted biases inherent in the LMs (*67*) could distort the results within feasible optimization limits. Moreover, although requiring intensive data collection from willing participants may currently ensure consent (*3*), advances in interindividual alignment technology could reduce this requirement (*68*, *69*). Therefore, it is imperative to establish regulations that promote the ethical use of these technologies (*70*) while ensuring explicit informed consent and safeguarding subjects’ mental privacy and autonomy in deciding which thoughts to disclose.

## MATERIALS AND METHODS

### Subjects

Six healthy individuals (S1: male, aged 37 to 38; S2: female, aged 37 to 38; S3: male, aged 33 to 34; S4: female, aged 35 to 36; S5: male, aged 29 to 30; and S6: male, aged 22 to 23) with normal or corrected-to-normal vision participated in the experiments. All were native Japanese speakers and non-native English speakers; S1 to S5 were proficient in English, while S6 had limited proficiency. All subjects provided written informed consent, and the study protocol was approved by the Ethics Committee of NTT (R03-004 and R03-009). The sample size was determined on the basis of prior fMRI studies with similar protocols (*7*, *65*). Data from each subject were collected over multiple scanning sessions spanning ~6 months. Experimental parameters and analytical pipelines were determined from a preliminary experiment with S1, who was exposed to the same stimuli multiple times, potentially influencing their brain responses.

### Visual stimuli

Visual stimuli consisted of 2196 short videos (all: 0.152 to 90.1 s, mean = 6.61 s, and median = 4.51 s; training: 0.152 to 90.1 s, mean = 6.70 s, and median = 4.62 s; test: 0.30 to 20.1 s, mean = 3.94 s, and median = 2.90 s) from a previous study (*36*) (https://goo.gl/forms/XErJw9sBeyuOyp5Q2). These videos covered diverse content (objects, scenes, actions, and events) and were resized to fit a 16° visual angle, maintaining the original aspect ratio. They were presented at the center of a gray background without sound. Sixteen duplicates were excluded to avoid redundancy, resulting in the final set of 2180 unique videos used in our experiment.

### Experimental design

We conducted two main experiments: a video presentation experiment and an imagery experiment (fig. S1). Visual stimuli were shown on a Liquid Crystal Display monitor at the rear of the fMRI scanner, and audio stimuli were delivered via S14 earphones (Sensimetrics). Each experimental session lasted up to 2 hours. Subjects were given adequate time for rest between runs (every 8 to 10 min) and could take a break or stop the experiment at any time. The total duration for both experiments was ~17.1 hours.

#### Video presentation experiment

The video presentation experiment included training sessions (60 runs) and test sessions (10 runs). Each run had 36 or 37 stimulus blocks and 3 or 4 randomly inserted evaluation blocks, averaging 695.2 s per run. Subjects spent ~11.8 hours on training sessions and 1.8 hours on test sessions.

In each stimulus block, videos shorter than 10 s were repeated until the total duration exceeded 10 s. Videos longer than 10 s were presented once, followed by less than 1 s of rest, making the block duration divisible by 1 s [repetition time (TR)]. Subjects viewed the stimuli without fixation to recognize details.

In each evaluation block, five descriptions (both in English and translated into Japanese) depicting the visual contents of videos were presented, and subjects were then asked to rate how consistent each description was with what they had perceived in the preceding stimulus block using two button boxes. The descriptions were taken from 20 captions for the preceding video, with occasional ones from other videos. Subjects rated the descriptions on a five-point scale or marked them with an “x” if they were unrelated (with higher scores indicating closer alignment with their perception; random initial score). Subjects completed the ratings at their own pace and proceeded to the next block by selecting “Proceed?” and pressing a button.

Each block was followed by a 2-s rest period, with 32- and 8-s rest periods at the beginning and end of each run, respectively. During the rest periods, the subjects were instructed to maintain fixation on a central spot, which consisted of a bull’s eye and crosshairs, to keep their attention focused on the screen.

In the training session, 2180 unique videos were each presented once in a pseudorandomized order. This order remained consistent for all subjects. Evaluated descriptions were also consistent for all subjects. In the test session, 72 videos from the last two runs of the training session were each presented five times, divided between two runs, and shown in a pseudorandom order within each run (see Materials and Methods’ “MRI data preprocessing” section for the data handling of the overlapping data in the training session).

#### Imagery experiment

The imagery experiment comprised 30 runs, each with 12 trials consisting of a preparation block, an imagery block, a video presentation block, and an evaluation block. Subjects were required to engage in recall-based visual imagery of one of the 72 videos presented during the test session of the video presentation experiment. Each run averaged 434.6 s, totaling ~3.6 hours per subject.

During a preparation block, a verbal description (both in English and translated into Japanese) of a target video was presented to subjects to prompt them to prepare mental imagery of the visual content of the target. The descriptions were selected from the set of 20 captions collected for each video and were consistent across trials and subjects. Subjects were encouraged to imagine all details, even those not explicitly described, to mimic the video presentation experiment. The description served as a guide to vividly imagine the complete visual content of the target. Subjects pressed a button when ready, and a beep sound with less than 1 s of rest signaled the start of the imagery period, aligning with the TR.

During the imagery period, subjects recalled the visual content of the target video with their eyes closed as if actually watching it. The imagery block duration matched the stimulus block duration in the video presentation experiment (repeatedly recalling for videos shorter than 10 s, once for videos longer than 10 s), with an additional 2 s to ensure full replay. A beep signaled the end of the imagery period, prompting subjects to open their eyes.

After the imagery block, the target video was presented as in the video presentation experiment, allowing subjects to compare their mental imagery with the actual video. This was followed by two 3-s blocks where subjects rated the accuracy and vividness of their imagery on a five-point scale. Subjects adjusted the score from its random initial setting using a button box in their right hand.

Each imagery, stimulus, and evaluation block was followed by a 2-s rest period, with 32- and 8-s rest periods at the beginning and end of each run. Subjects maintained fixation on a central spot, as in the video presentation experiment.

Before the imagery experiment, subjects practiced associating each target video with its verbal description, viewing the pairs during interrun rest periods to aid memory. The 72 videos were randomly distributed among six runs, with each set of six runs containing all videos in a pseudorandom order.

#### Retinotopy and functional localizer experiments

In addition to the main experiments, we conducted a retinotopy experiment and three functional localizer experiments (visual category, MT+, and language area localizers) to delineate visual areas and localize regions of interest (ROIs).

##### Retinotopy

We followed the Human Connectome Project 7T Retinotopy Dataset protocol (*71*) using dynamic colorful textures through moving apertures (wedge, ring, and bar) in eight 300-s runs. This identified retinotopic maps (V1, V2, V3, V3A, V3B, hV4, and V7) on cortical surfaces using fsfast retinotopy analysis [Freesurfer (*72*)] and population receptive field analysis (code is available at https://kendrickkay.net/analyzePRF/) (*73*).

##### Visual category localizer

Following the fLoc protocol (*74*) (stimuli and code are available at http://vpnl.stanford.edu/fLoc/), we presented images from word, body, face, place, and object categories in eight 300-s runs (48 blocks each, 6 s per stimulus or blank, with 6-s initial and final rests). Additional intact and scrambled object conditions were included to localize object-selective areas (original images are available at tarrlab; https://sites.google.com/andrew.cmu.edu/tarrlab/stimuli). The contrasts between word/body/face/place and others (from these four categories) were used to define visual category-selective areas [word: visual word form area (VWFA) and occipital word form area (OWFA); body: extrastriate body area (EBA) and fusiform body area (FBA); face: fusiform face area (FFA) and occipital face area (OFA); place: parahippocampal place area (PPA), occipital place area (OPA), and medial place area (MPA) consisted of the retrosplenial cortex and parieto-occipital sulcus]. The contrast between intact and scrambled objects was used to define an object-selective area [lateral occipital complex (LOC)].

##### MT+ localizer

Following Tootell *et al.* (*75*), we presented random dot stimuli in three conditions (moving, dynamic, and static) in four 232-s runs (13 blocks each, 12 s per stimulus, with 12-s initial and final rests). The contrast between moving and dynamic/static conditions defined the visual motion area MT+.

##### Language area localizer

We followed protocols by Fedorenko *et al.* (*76*) and Scott *et al.* (*77*), modifying to include both visual and auditory stimuli and to use Japanese stimuli in each of eight 358-s runs (19 blocks each, 18 s per stimulus or 14-s blank, with 14-s initial and final rests). Subjects read sentences or nonword sequences and listened to intact or degraded auditory passages. The contrasts between sentence/intact and nonword/degraded conditions defined language-sensitive areas in the temporal and frontal cortices.

Voxels from V1, V2, and V3 were combined as the LVC; voxels from VWFA, OWFA, EBA, FBA, FFA, OFA, PPA, OPA, MPA, LOC, and MT+ were combined as the HVC; and voxels from temporal and frontal language areas were combined as the language network. Overlapping voxels with LVC were excluded from HVC.

### MRI acquisition

MRI data were collected using a 3.0-Tesla Siemens MAGNETOM Prisma scanner located at the WPI-IRCN Human fMRI Core, the University of Tokyo Institutes for Advanced Studies. An interleaved T2*-weighted gradient-echo echo-planar imaging scan was performed to acquire functional images covering the entire brain [TR: 1000 ms; echo time (TE): 30 ms; flip angle: 65°; field of view (FOV): 192 mm by 192 mm; voxel size: 2 mm by 2 mm by 2 mm; slice gap: 0 mm; number of slices: 72; multiband factor: 6]. T1-weighted (T1w) magnetization-prepared rapid acquisition gradient-echo fine-structural images of the entire head were also acquired [TR: 2000 ms; TE: 1.97 ms; inversion time (TI): 900 ms; flip angle: 10°; FOV: 256 mm by 256 mm; voxel size: 1.0 mm by 1.0 mm by 1.0 mm].

### MRI data preprocessing

For anatomical data, we first used SPM12 to preprocess each of the T1w anatomical images of individual subjects for bias-field correction and for redefining its origin and orientation to be set on the anterior commissure and the anterior commissure–posterior commissure line, respectively. Cortical surface meshes were generated from the processed T1w images using Freesurfer (version 7.3.2) (*72*) with manual corrections for anatomical segmentations. Analytical results were visualized on flattened cortical surfaces, created by making relaxation cuts in each hemisphere, with functional data aligned and projected using Pycortex (*78*).

For the functional data from each run, we performed the MRI data preprocessing through the pipeline provided by fMRIPrep (version 20.2.7) (*79*). First, a BOLD reference image was generated using a custom methodology of fMRIPrep. A field map (B0-nonuniformity map) estimated on the basis of a phase-difference map was used to estimate susceptibility distortion and to correct the BOLD reference for a more accurate coregistration with the anatomical reference. The BOLD reference was then coregistered to the T1w reference using bbregister (FreeSurfer; version 7.3.2), which implements boundary-based registration (*80*). BOLD runs were slice-time corrected using 3dTshift from AFNI 20160207 (*81*), and the BOLD time series were resampled onto their original, native space (2 mm–by–2 mm–by–2 mm voxels) by applying a single, composite transform to correct for head motion, and susceptibility distortions using antsApplyTransforms from ANTs (version 2.3.3) with Lanczos interpolation.

To create data samples, we first discarded the first 8-s scans of the preprocessed BOLD signals from each run to avoid MRI scanner instability. We then regressed out nuisance parameters from each voxel amplitude for each run, including a constant baseline, a linear trend, and 24 head-motion parameters (three rotations, three translations, their temporal derivatives, and quadratic terms) and 12 global signals (mean amplitudes within cerebrospinal fluid, white matter, gray matter, their temporal derivatives, and quadratic terms). The data samples were temporally shifted by 4 s to account for hemodynamic delays, despiked to reduce extreme values (beyond ±3 SD for each run), and averaged within each stimulus and imagery block. Last, each voxel’s amplitude was *z*-scored within each run to eliminate potential nonstationarities and scanner-specific biases.

For data from the training session of the video presentation experiment (training perception data), we discarded samples from the last two runs, in which videos used in the test session and the imagery experiment were presented, to ensure generalization to new stimuli. For test data from the video presentation experiment (test perception data) and the imagery experiment (test imagery data), we averaged samples of identical video clips (five repetitions) to increase the signal-to-noise ratio of the fMRI signals unless otherwise stated.

### fMRI brain activity data from Natural Scenes Dataset

We additionally used the Natural Scenes Dataset (NSD) (*39*), which contains high-resolution fMRI signals from eight subjects measured across 30 to 40 recording sessions. Our analysis focused on data from four subjects (subjects 1, 2, 5, and 7) who completed all 40 sessions. We analyzed image-induced fMRI activity, while these subjects viewed images from MS-COCO (*82*). We used preprocessed fMRI signals in 1.8-mm native volume space corresponding to “nsdgeneral” brain areas, which included ~15,000 voxels per subject in the posterior cortex responsive to visual stimuli. Using the code from Ferrante *et al.* (*18*) (https://github.com/enomodnara/BrainCaptioning), we constructed fMRI data samples of image-induced activity corresponding to 8859 images for training and 982 images for testing. Because these stimulus images were presented up to three times, we averaged the fMRI signals from multiple trials to enhance the signal-to-noise ratio. For the analysis with NSD, we used the API (application programming interface) of ChatGPT (GPT-4o mini; https://chat.openai.com/; prompt: “Please minimally proofread the following set of image captions. Captions:”) to proofread and refine the captions, as the MS-COCO captions often contain typos and grammatical errors.

### Caption annotation for visual stimuli

We used Amazon Mechanical Turk to collect written captions for stimulus video clips, following the procedure used for Microsoft COCO Captions (*83*). Multiple workers viewed each video to provide a detailed sentence (more than eight words) describing the visual content. The captions were manually checked for quality and proofread with the assistance of ChatGPT (GPT-3.5; https://chat.openai.com/; prompt: “Proofread the following:”) to correct typos and remove incorrect or unintelligible sentences. We collected 20 unique captions per video, matching the number in the MSR-VTT dataset (*84*). The collected video captions are available from our repository (https://github.com/horikawa-t/MindCaptioning).

### Feature computation by DNN models

We used DNN models pretrained for language (e.g., DeBERTa-large) or vision (TimeSformer) (*54*) tasks to compute semantic and visual features, respectively. In addition, we used the image encoder of a multimodal model (CLIP) (*56*) to compute visuo-semantic features. To mitigate biases arising from baseline differences across model units, we applied *z*-score normalization to the feature values using means and standard deviations (SDs) estimated from respective training data for each analysis.

#### Semantic features

To extract semantic features from video captions, we used 42 pretrained LMs (available at Hugging Face’s Transformers library, version 4.30.2) (*85*). These models cover a range of architectures (e.g., BERT and GPT-2) and sizes (e.g., base, large, and xlarge). Each input sequence was tokenized and processed by an LM to produce vector embeddings for each token across multiple layers. Following Reimers and Gurevych (*86*), we averaged the embeddings over tokens, excluding special tokens (e.g., <cls> token), in each layer. The averaged embeddings from multiple layers were used as semantic features for the input sequence. For each video, we computed semantic features for 20 annotated captions and averaged them to construct the final semantic features for the video.

The 42 LMs used in the present study were based on the following model families: BERT (*35*), RoBERTa (*36*), DeBERTa (*37*), ALBERT (*87*), OpenAI-GPT (*88*), GPT-2 (*89*), Sentence GPT (*90*), XLNet (*91*), DistilBERT (*92*), DistilGPT2 (*85*), T5 (*93*), BART (*94*), CTRL (*95*), XLM (*96*), XLM-RoBERTa (*97*), ELECTRA (*98*), and CLIP (*56*) text encoder. See fig. S8 for the full list of the LMs.

We hypothesized that an LM closely aligned with the human brain would provide more effective intermediate representations for translating visual semantic information in the brain into text. We thus performed a cross-validation encoding analysis using semantic features from each of the 42 LMs within the training perception data. On the basis of the results of the validation analysis (fig. S2A), we selected the DeBERTa-large model, which demonstrated the highest performance.

To construct semantic features without structured semantic information (fig. S3C), we used captions with randomly shuffled word orders. For each caption, we created up to 1000 word-order shuffled variants, computed their semantic features, and averaged these features across all the variants. These features were then averaged across 20 captions for each video to obtain semantic features for each video.

#### Vision model features

To extract visual features from video stimuli, we used a TimeSformer model (*54*) (model and code are available at https://github.com/facebookresearch/TimeSformer) pretrained for object and action recognition using ImageNet (*99*) and Kinetics-400 (*100*). This model has demonstrated high performance in predicting video-induced brain activity (*101*). For each video, we resized its spatial size to 224 pixels while preserving the aspect ratio and selected frames at intervals of 32. If the video had fewer than eight temporal positions at 32-frame intervals, we uniformly selected eight frames to cover the entire video length. We computed feature vectors for each layer from these frames and averaged them over the spatial dimension within each layer. This procedure was repeated for three spatial crops (left-center-right or top-center-bottom). Last, we averaged the visual features over the temporal dimension and the three spatial positions, resulting in 768-dimensional features for each of the 12 layers.

#### Visuo-semantic model features

To extract visuo-semantic features from video stimuli, we used the image encoder of CLIP (ViT-B/16) developed by OpenAI (available at Hugging Face’s Transformers library, version 4.30.2) (*85*). We primarily used this model to replicate the nonlinear image captioning–based decoding method, known as brain captioning (*18*). Because this approach was originally designed for image-induced brain activity, we adapted it for video-induced brain activity by averaging features across time (or multiple frames). Specifically, we extracted features from the final layer of the vision encoder for each frame using the default preprocessing and concatenated the resulting 768-dimensional embeddings from 197 tokens, including the [CLS] token and 196 patch tokens. We then averaged these frame-wise features, resulting in a 151,296-dimensional visuo-semantic feature vector for each video.

### Voxelwise encoding modeling analysis

We performed voxelwise encoding modeling analysis by constructing encoding models that predict signal amplitudes of individual voxels from a feature vector in each model layer using the L2 regularized linear regression algorithm (ridge regression). The analysis was performed using both cross-validation and generalization approaches. In the cross-validation analysis, we used sixfold cross-validation on the training perception data (58 runs divided into five sets of 10 runs and one set of 8 runs). In the generalization analysis, we trained models on all of the training perception data and tested them on the test perception data (fig. S8). We evaluated the model performance of each voxel by calculating Pearson correlation coefficients between measured and predicted brain activities of that voxel.

The regularization parameters of ridge regression were determined separately for each layer, model, and subject by considering the performance of all voxels on the respective training data. Models for individual voxels were trained using 10 possible regularization coefficients (log spaced between 10 and 10,000). The regularization parameters that produced the maximal model performance (mean correlation coefficients averaged across all voxels) on the training data were used for predictions on the test data. In the cross-validation analysis, we used a fivefold cross-validation (inner loop) nested within a sixfold cross-validation (outer loop). In the generalization analysis, we used sixfold cross-validation on the training perception data.

For each model, predictions from multiple layers were integrated by selecting the best layer for each voxel based on model performance in the training data. In each fold of the cross-validation analysis, we determined the best layer per voxel from the sixfold nested cross-validation loops (cf. Fig. 3F). We aggregated predictions from these best layers for each left-out set to construct final predictions for all data samples. In the generalization analysis, the best layer per voxel was determined from the sixfold cross-validation on the entire training perception data, and predictions from the best layers were used for the test perception data.

Encoding accuracies of semantic and visual models were compared using slope angles of the best linear fit estimated by Deming regression (*102*), which accounts for observation errors on both axes. The slopes were converted to angles and then subtracted from 45° to obtain deviations from parity (Fig. 3E).

### Feature decoding analysis

We performed feature decoding analysis by constructing a set of L2 regularized linear regression models (decoders) that predict feature values for each layer of each model from fMRI activity patterns (one decoder for each model unit). Both cross-validation and generalization analyses were performed to produce predictions for all training perception data samples and the test perception and imagery data samples. Decoders were trained using whole-brain fMRI voxel patterns (unless otherwise stated), selecting up to 50,000 voxels that were best predicted by the target feature set in cross-validation (or nested cross-validation) encoding analysis on the respective training data. Performance was evaluated by calculating Pearson correlation coefficients between feature values computed by an LM and predicted from the brain for each model unit.

In the cross-validation analysis, we used sixfold cross-validation on the training perception data, with model training and the regularization parameter estimation conducted using a fivefold cross-validation procedure nested within the sixfold cross-validation, similar to the encoding analysis. In the generalization analysis, models were trained on all the training perception data and tested on the test perception and test imagery data, with regularization parameters determined using a sixfold cross-validation within the entire training perception data. Regularization parameters of the ridge regression models were optimized on the basis of model performance on the respective training data, with coefficients estimated separately for each layer, model, and subject considering the performance of all units.

### Text generation analysis

To generate descriptive text based on a set of semantic features (target features), we conducted an iterative optimization of descriptions, in which semantic features of candidate descriptions were progressively aligned with target features through the iterative replacement and interpolation of tokens (referred to as “words” for simplicity) within the candidate descriptions. Each step of the optimization process consisted of three stages: masking, unmasking, and candidate selection.

In the masking stage, for each of the current candidate descriptions (e.g., “metal shapes”), we first generated an exhaustive list of masked candidates by replacing each word or a sequence of words (three words at the maximum) with a mask token (e.g., “<MASK> shapes,” “metal <MASK>,” and “<MASK>”) or interpolating a mask token between words or at the top or bottom of the description (e.g., “metal <MASK> shapes,” “<MASK> metal shapes,” and “metal shapes <MASK>”). This masking procedure was repeatedly applied to the newly generated masked candidates (two times at the maximum) to generate masked candidates with multiple masks (e.g., “<MASK> <MASK> shapes” and “metal <MASK> <MASK>”). From all the generated masked candidates for each original candidate, we randomly selected five masked candidates to be processed in the next unmasking stage.

In the unmasking stage, we used an LM pretrained for MLM (not necessarily the same as the model used for the feature computation) to generate alternative words to fill in the masks in the masked candidates within the context of surrounding words. For each mask within a masked candidate description, we generated five alternative words for the mask to create five new candidates by random sampling from a categorical distribution likelihood estimated by the MLM model. In cases where a masked candidate had multiple masks, we processed the masks sequentially from the top until all masks were updated. These procedures yielded five new candidates from one masked candidate. In the main analysis, we used the pretrained RoBERTa-large model (vocabulary size: 50,265; subword segmentation based on byte pair encoding) for the guide of the text generation because this model consistently demonstrated stable performance in optimizing descriptions in a validation analysis (fig. S2B).

In the candidate selection stage, we computed semantic features of all new and original candidate descriptions using an LM from which the target features originated. We then calculated Pearson correlation coefficients between those candidate features and target features for all layers and averaged those correlations over layers to score the candidate descriptions. To enhance the conciseness of generated descriptions, reduce the computational cost of handling long descriptions, and avoid overfitting to noises on brain-decoded features, we added an exponential penalty to the length of candidate descriptions as described by $s=\frac{r}{l^{\alpha }}$ , where s is the similarity score used to rank candidates, r is the mean correlation coefficient between candidate features and target features averaged across layers, l is the length (or the number of tokens) of the candidate, and α is the parameter for the length penalty. We chose the parameter α = 0.1 based on the validation analyses using six possible penalty parameters (0, 0.05, 0.1, 0.15, 0.2, and 0.25) with a randomly selected subset of 50 samples from the training perception data (fig. S2, C to E). After computing similarity scores for all candidates, we ranked them and selected the top five candidates to proceed with further optimization.

During each step of the optimization process, the maximum search width was 130 (five new candidates for each of the five masked candidates derived from five original candidates, in addition to the five original candidates themselves).

We repeated these optimization stages 100 times, and the obtained description was taken to be the text describing the semantic information represented in the target features, or the brain. Because the masking and unmasking stages involve randomness in the optimization process, to avoid local optima, we repeated the same process five times for each data sample to select the description showing the highest similarity scores with the target features as the final prediction. For all the analysis, we began the optimization process from a noninformative initial state (i.e., unknown token, <unk> for the tokenizer of the RoBERTa-large) to avoid incorporating any prior assumptions for description generation.

Unlike autoregressive LMs with causal attention often used for linguistic information decoding (*3*, *4*), MLM model with bidirectional attention has the substantial advantage of incorporating contextual information from all surrounding words (*35*). This characteristic makes our optimization process more suitable for decoding visual information, which lacks specific directionality, in contrast to linguistic information.

### DB-search–based description prediction analysis

Among the notable strengths of our method are its flexibility in optimizing descriptions at the word (or token) level and its ability to generate word sequences that do not currently exist in the databases. To assess its effectiveness, we used the DB-search method (*17*) to set a baseline performance for description prediction from the brain. This analysis involved searching for captions with the highest feature correlations with target brain-decoded features from large databases of image and video captions [MS-COCO (*82*), GCC (*103*), and MSR-VTT (*84*)]. We computed semantic features for all captions (~4.1 M) using the DeBERTa-large model. For each fMRI data sample, we predicted semantic features at multiple layers using trained decoders and computed correlation coefficients between the decoded features and features computed from all database captions. The caption with the highest mean correlations across all layers was selected as the prediction for the sample. The brain-decoded features used in this analysis were the same as those used in the main analysis (i.e., mind captioning; e.g., Fig. 2).

We also performed the same analysis on fMRI data from the NSD (fig. S7) (*39*), using candidate captions from the GCC image database (*103*) to match the experimental conditions used in the previous study (*17*).

### Nonlinear image captioning–based description prediction analysis

Another strength of our method is that it does not require training an additional module, such as nonlinear captioning models, by directly optimizing word sequences to match brain-decoded features. To examine the effectiveness of this aspect, we compared its performance with another brain-to-text decoding approach based on a nonlinear image captioning model (brain captioning) (*18*). We implemented this method using the code from the authors’ repository (https://github.com/enomodnara/BrainCaptioning). This approach uses an independently trained image captioning model, the Generative Image-to-text Transformer (GIT) (*57*), which generates descriptions by processing visuo-semantic features extracted from CLIP (*56*). We computed visuo-semantic features for all stimuli (videos or images) using CLIP. For each fMRI data sample, we predicted visuo-semantic features from the final layer using trained decoders and inputted these features into GIT to produce a description.

### Evaluation of the similarity between generated descriptions and references

We used multiple metrics to evaluate the similarity of generated descriptions to reference captions annotated to the viewed or recalled videos. These metrics include Pearson correlation (feature correlation), BLEU, METEOR, ROUGE-L, CIDEr, and three variants of BERTScore. For each predicted description, we computed scores for each metric against 20 reference captions of the corresponding video, selecting the highest score as the final score. BLEU and METEOR were computed using the NLTK toolbox (*104*), while ROUGE-L and CIDEr were computed using code from https://github.com/salaniz/pycocoevalcap. BERTScore was computed using code from https://github.com/Tiiiger/bert_score.

#### Feature correlation

We defined the feature correlation as the mean of Pearson correlation coefficients between semantic features of a reference caption or target features and those of a generated description averaged across multiple layers. We computed semantic features using the same LM as in the decoding analysis, unless otherwise stated. Feature correlation was used in all analyses where the similarity metric was not explicitly specified.

#### BLEU

BLEU computes the precision scores by comparing predicted *n*-grams with reference captions while considering a brevity penalty. We used the 4-gram variant (BLEU-4) with a smoothing method (*105*).

#### METEOR

METEOR computes scores based on unigram matching in predicted and reference sequences using precision and recall while considering word variations such as stemming and synonymy.

#### ROUGE-L

ROUGE-L computes scores by emphasizing recall, measuring the overlap of words between the prediction and reference, and primarily focusing on their longest shared sequence.

#### CIDEr

CIDEr measures the similarity between predicted descriptions and references through *n*-gram–based comparison while considering consensus across multiple references.

#### BERTScore

BERTScore computes similarity using contextualized embeddings from individual tokens by a bidirectional transformer LM. We used the 17th layer of the RoBERTa-large model with baseline rescaling to compute scores (default of the official implementation) and evaluated three variants: P (precision), R (recall), and F1. To evaluate the token-wise precision of generated descriptions, we used the precision (P) weighted by the inverse document frequency (IDF) estimated from captions in multiple databases (MSCOCO, GCC, and MSR-VTT) and captions collected in this study. The IDF-weighted P was only used to highlight tokens with high precision in generated descriptions but not used in quantitative evaluations.

On the basis of these metrics, we evaluated the discriminability of the generated descriptions. For each description, we calculated similarity scores between the generated description and reference captions of the corresponding viewed or recalled videos (correct) and irrelevant videos (incorrect; *n* = 2179). Discriminability was defined as the difference between the score for the correct video and the mean score averaged across all incorrect videos.

### Text-based video identification analysis

To evaluate description generation performance, we performed a video identification analysis based on the similarity between generated descriptions and reference captions using multiple metrics. For each data sample, we computed similarity scores between the generated description and all reference captions of all videos using a specific metric. We compared the similarity scores to the correct reference captions (20 captions for the target video) with those to incorrect reference captions (43,580 captions for 2179 irrelevant videos). For the analysis with feature correlation and BERTScore, we used mean similarity scores averaged across multiple captions per video. For BLEU, METEOR, ROUGE-L, and CIDEr, we used the highest matching scores among multiple captions per video. The analysis was conducted with varying numbers of candidates, ranging from 2 (chance level = 50%) to 100 (chance level = 1%), selecting the video with the highest similarity score as the prediction. Accuracy was defined as the proportions of correct video identification. For two candidates (one correct and the other incorrect), we performed identification for all combinations of correct and incorrect candidates. For more than two candidates, we randomly selected incorrect candidates, repeating the analysis 100 times to estimate the mean accuracy averaged across repetitions.

### Feature-based video identification analysis

To evaluate the generalizability of feature decoders trained on stimulus-induced brain activity for decoding imagery-induced activity, we performed a feature-based video identification analysis and compared accuracies between perception and imagery conditions (cf. Fig. 4E). The analysis was conducted separately for each model and layer by comparing brain-decoded feature vectors with feature vectors derived from video captions or visual stimuli using three types of models (visual, visuo-semantic, and semantic). For each data sample, we calculated Pearson correlation coefficients between the decoded feature vector and the model-derived feature vectors of all candidate videos (*n* = 2180). Identification was performed in a pairwise manner by determining, for each pair consisting of the correct video and one of the 2179 incorrect videos, whether the correlation with the correct video was higher (chance level = 50%). Accuracy was defined as the proportion of pairs in which the correct video had a higher correlation than the incorrect one. For each model layer, we assessed the generalizability from perception to imagery by measuring the angle between the parity line (i.e., the diagonal line indicating equal accuracy for the two conditions) and a line connecting the chance level to the observed accuracies for the perception and imagery conditions. Smaller angles indicate stronger generalizability.

### Evaluation of the relational information in generated descriptions

To assess whether the generated descriptions accurately represented visual relationships among individual components in viewed or recalled videos, we evaluated the effect of shuffling word orders of generated descriptions on discriminability and video identification performance. For example, distinctions such as a bird eats a snake versus a snake eats a bird or some grass in a mug versus a mug in some grass (*43*) are sensitive to word order and thus indicative of relational structure. Disrupting this word order through shuffling can therefore help reveal whether such structured information is accurately described in the generated text. We randomly shuffled the word order of each generated description to create word-shuffled variants. The shuffling was performed at the word level, not the token level, to maintain minimal coherence within individual words. For each original description, we created up to 1000 shuffled variants by shuffling all words or only nouns, excluding descriptions with only one noun from the noun-shuffling analysis. Nouns were identified using part-of-speech tagging with spaCy (version 2.2.4) (*106*). We then computed semantic features for these shuffled descriptions.

If generated descriptions accurately capture visual relations among individual components in videos, then we should observe higher similarity to correct captions with the original descriptions compared to the shuffled ones while maintaining differences with irrelevant captions. To examine this, we computed feature correlation scores between reference captions and both original and shuffled descriptions to examine whether the original descriptions exhibited higher discriminability (e.g., Fig. 2F, right). Given the varying levels of sentence structure disruption among the shuffled descriptions, we also conducted the same analysis using the least-disrupted shuffled sentences to rigorously assess the superiority of the original descriptions. Specifically, we selected the shuffled sentence with the highest pseudo–log-likelihood score—a fluency (or linguistic acceptability) metric computed by MLM scoring (*44*)—from 1000 shuffled variants for each generated description (e.g., fig. S4F). We used an adapted metric proposed by Kauf and Ivanova (*45*). In addition, to quantify the impact of shuffling on identification accuracy, we performed video identification analysis using both original and shuffled descriptions (e.g., Fig. 2F, left).

We also examined whether the word order of generated descriptions was unduly influenced by the MLM model used to support the text generation, potentially diverging from the information represented in the brain. We reasoned that if a generated description closely matches the brain representation and contains semantic information uniquely depicted by the generated word order—rather than alternative arrangements of the same words—then brain-decoded features should exhibit higher similarity to features of the generated description than features of shuffled variants. To test this, we computed feature correlation scores between target brain-decoded features and both original and shuffled descriptions to see whether the original exhibited greater scores. To consider the degree of meaning changes introduced by shuffling, we also computed feature correlation scores between original and shuffled descriptions, defining correlation distance as one minus the feature correlation (Fig. 2G and fig. S4G).

### Evaluation of the diversity in generated descriptions

To evaluate the diversity of generated descriptions (fig. S5, C and D), we used Self-BLEU (*107*)—a metric assessing the diversity of the generated text data—to compute the sentence (dis)similarity either across subjects for each video or across videos for each subject. For each video/subject, we can compute a BLEU score by regarding one description from a subject/video as a hypothesis and descriptions from the other subjects/videos as references. To evaluate the diversity across subjects/videos, we computed BLEU scores for every generated description from different subjects/videos and defined the average BLEU scores across subjects/videos as the Self-BLEU of the video/subject, respectively. A higher Self-BLEU score indicates less diversity in the descriptions generated for the video/subject. In this analysis, we used the same analytical settings as we did when using the BLEU for the similarity evaluation (BLEU-4 with smoothing).

### Evaluation of the consistency between generated descriptions and subjective perception

The reference captions in this study were collected from subjects in an independent online experiment, not from our fMRI subjects. Therefore, not all captions may precisely match the subjective perception of our fMRI subjects, although they were used as “correct” references in the evaluation. To examine whether the generated descriptions from video-induced brain activity were consistent with the subjective perceptions of individual subjects, we investigated the relationship between subjective ratings from the video presentation experiment and the similarity of generated descriptions to the evaluated captions. We focused on 212 videos evaluated during the training perception data collection, analyzing descriptions generated from the cross-validation decoding analysis using feature correlations as the similarity metric. For each generated description, we computed feature correlations against five evaluated captions, yielding 1060 scores per subject. These scores were classified according to individual ratings to explore whether higher-rated captions had higher scores. In addition, we calculated Pearson correlation coefficients between the feature correlation scores and subjective ratings to determine the presence of positive correlations (fig. S5E).

### Illustrations

Because of copyright restrictions, the images shown here are not actual frames from the video stimuli used in our experiments. Instead, they are schematic illustrations manually created by a professional scientific illustration company (Medical Education, Tokyo, Japan), based on the captions of the original videos. All illustration copyrights have been transferred to the author.

### Statistical analysis

Statistical analysis was performed individually unless otherwise stated, with results from six subjects considered as replications (*108*). We reported quantitative results for each subject and averages across subjects, except for the validation analysis (fig. S2, C to E). To account for multiple comparisons, we used the Benjamini-Hochberg method (*109*) to control the FDR and provided this information where applicable. Statistical significance between results of pretrained and untrained MLM model was tested using a one-tailed Wilcoxon signed-rank test on feature correlations of generated descriptions after 100 optimization iterations (*n* = 72; fig. S4A).

Discriminability based on generated descriptions was also tested using a one-tailed Wilcoxon signed-rank test (*n* = 72; e.g., fig. S4B). The effect size of discriminability (e.g., Fig. 2D) was computed by calculating similarity scores between a generated description and both sets of correct (*n* = 20) and incorrect captions (*n* = 43,580). These scores were averaged separately for each set. These averaged scores for correct and incorrect sets from all test samples (*n* = 72) were used to estimate means and SDs for computing Cohen’s *d* for discriminability.

Video identification analysis results were presented with a 95% confidence interval (CI) across samples (*n* = 72) to determine whether the mean accuracy exceeded the chance level (e.g., Fig. 2E).

We used one-tailed Wilcoxon signed-rank tests to evaluate the impact of word-order shuffling on discriminability (*n* = 72; e.g., Fig. 2F) and the diversity differences in generated descriptions (*n* = 72 for self-BLEU across subjects and *n* = 6 for self-BLEU across videos; fig. S5, C and D).

Correlations between discriminability and encoding accuracy across multiple LMs were evaluated using one-tailed *t* tests after Fisher’s *z* transform (*n* = 42; fig. S8B). The correlation between layer depth and both discriminability and its drop caused by shuffling was tested using a one-tailed *t* test (fig. S9).

The variability of the generalization angle was estimated using a jackknife resampling procedure. Specifically, one of the 72 samples—defined as the identification accuracy for each pair of viewed and imagined videos—was sequentially left out, and the angle was computed from the remaining 71 samples. The standard error was then estimated from the distribution of these leave-one-out angle values (e.g., Fig. 4E).

To evaluate the similarity between the generated descriptions and rated captions, we pooled results from all six subjects to ensure sufficient data samples to detect differences while also accounting for variations in the number of samples across rating levels and subjects (fig. S5E). Differences in feature correlations across subjective ratings were tested using one-tailed *t* tests after Fisher’s *z* transform. Correlation between ratings and feature correlations between generated descriptions and rated captions were tested using a one-tailed *t* test after applying Fisher’s *z* transform (*n* = 6360). Interactions between text generation methods and ratings were tested using analysis of variance (ANOVA).

For encoding analysis, the correlation between measured and predicted fMRI signals for each voxel was tested using a one-tailed *t* test after Fisher’s *z* transform (*n* = 2108; Fig. 3, A and B). Mean encoding accuracy within each brain area was presented with a 95% CI across voxels (Fig. 3D). Comparisons between semantic and visual encoding models were based on the slope angles of linear fits, converted to deviations from parity (Fig. 3E). Statistical significance of differences in the best layers exhibiting highest encoding accuracy was tested using a one-tailed Wilcoxon rank sum test (Fig. 3F).
