# Supplementary material for: Mind captioning: Evolving descriptive text of mental content from human brain activity
Source: Sci Adv. 2025 Nov 5;11(45):eadw1464. doi: 10.1126/sciadv.adw1464 (PMC12588295; doi:10.1126/sciadv.adw1464)
Supplement: Supplementary file 1 — Figs. S1 to S14 [file sciadv.adw1464_sm.pdf]

Science Advances
AAAS

Supplementary Materials for

Mind captioning: Evolving descriptive text of mental content from human brain activity

Tomoyasu Horikawa

Corresponding author: Tomoyasu Horikawa, horikawa.t@gmail.com

Sci. Adv. **11**, eadw1464 (2025)
DOI: 10.1126/sciadv.adw1464

This PDF file includes:

Figs. S1 to S14

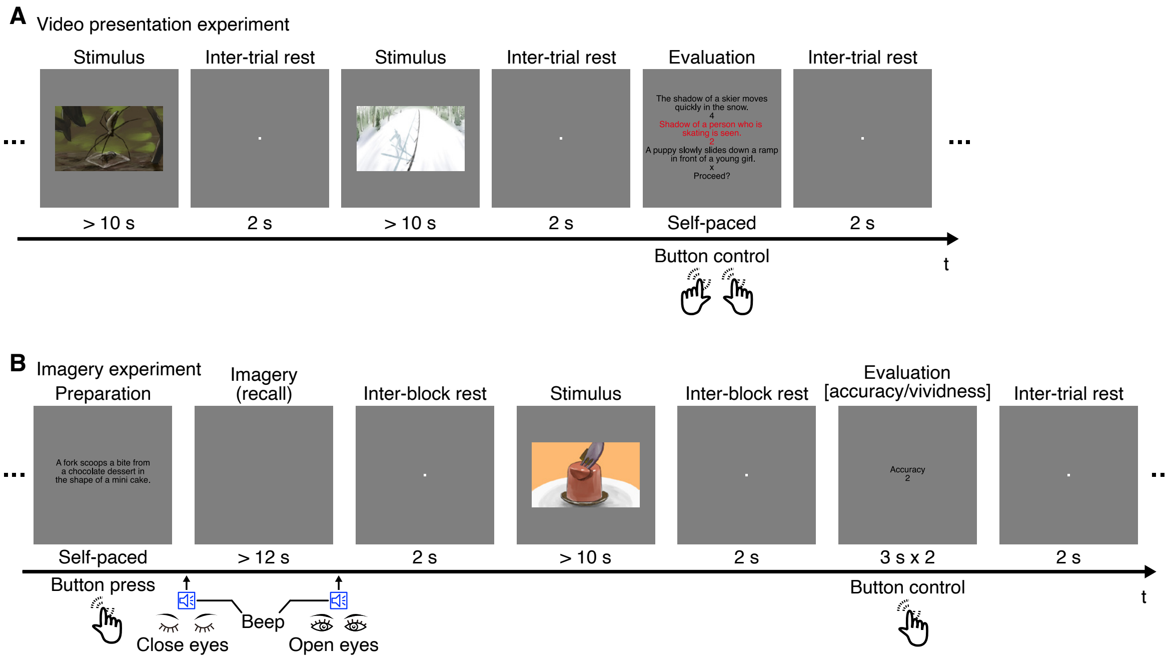

Fig. S1. Overview of experiments.
We conducted two types of experiments. (**A**) Video presentation experiment (training and test sessions). Subjects were allowed to view videos without fixation. Occasionally, we presented five descriptions depicting the visual content of the preceding video to the subjects, asking them to rate the consistency between each description and their subjective perception. We used these ratings to evaluate the consistency between descriptions generated from the brain and the subjective perception of each individual (fig. S5E). (**B**) Imagery experiment. Subjects were well-trained to associate descriptions and videos before the experiment. They were required to visually imagine (recall) a video based on a description of the target video with their eyes closed according to beeps. Each imagery block was followed by a stimulus block, during which the target video was presented to allow the subjects to confirm the validity of their imagery. An evaluation block followed, asking the subjects to evaluate the accuracy and vividness of their imagery. Data samples were constructed by averaging fMRI volumes during each stimulus/imagery block. Training data contained samples for 2,108 videos, each presented once. Test and imagery data contained samples for the same 72 videos (not used during training), averaged across five repetitions.

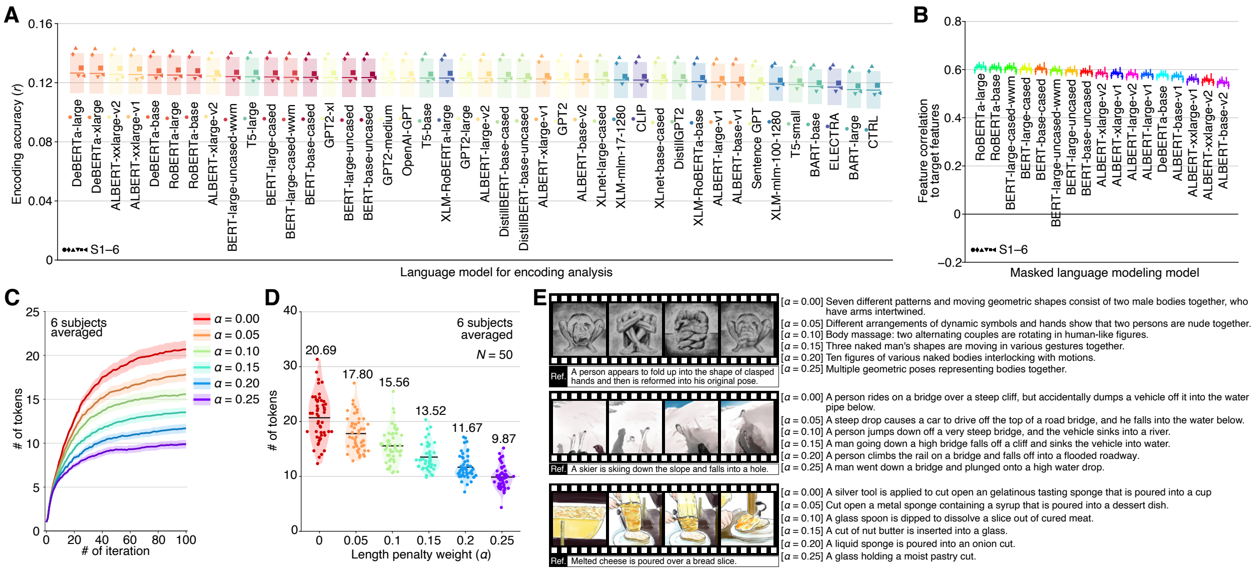

Fig. S2. Validation of model and parameter determinations.

Validation analyses were performed using cross-validation within the training perception data to determine LMs used in feature decoding and text generation analyses and a length penalty parameter. (**A**) Encoding accuracy from different LMs (averaged across voxels). We hypothesized that an LM with better performance in predicting brain responses to visual stimuli would provide more effective intermediate representations for translating visual semantic information in the brain into text. Accordingly, we performed an encoding analysis using features from each of the 42 LMs and found that the DeBERTa-large model consistently exhibited the best performance. Therefore, we used it in the main analysis. (**B**) Feature correlations between target decoded features and features of descriptions generated with different MLM models. Different MLM models were used in the text generation analysis (the DeBERTa-large model was used for feature decoding and evaluation). All models exhibited reliable performance in generating descriptions aligned with target decoded features. We decided to use the RoBERTa-large model, as it exhibited the best performance. (**C**) The number of tokens in generated descriptions through optimization. (**D** and **E**) The number of tokens in generated descriptions (D) and the example descriptions (E) obtained with varying strength of length penalty. We evaluated the similarity between target brain-decoded features and features of candidate descriptions based on Pearson correlation coefficients with an exponential penalty to the length of candidate descriptions (see Materials and Methods for details). To determine the length penalty parameter, we performed a validation analysis with a randomly selected subset of 50 samples from the training perception data using six possible penalty parameters (0, 0.05, 0.1, 0.15, 0.2, and 0.25). While the length of generated descriptions varied depending on the length penalty, accurate descriptions of viewed content were consistently generated. We decided to use $\boldsymbol{\alpha} = 0.1$, as the mean length of generated descriptions with this parameter (mean = 15.56) was comparable with that of reference captions in the training perception data (mean = 15.94). In (B−E), the text generation analysis was performed for 50 videos randomly selected from the training perception data (DeBERTa-large; decoded from whole brain activity). Shades in (A, B) indicate 95% C.I. across subjects ($N = 6$). Error bars in (A, B) and shades in (C) indicate 95% C.I. across samples ($N = 50$).

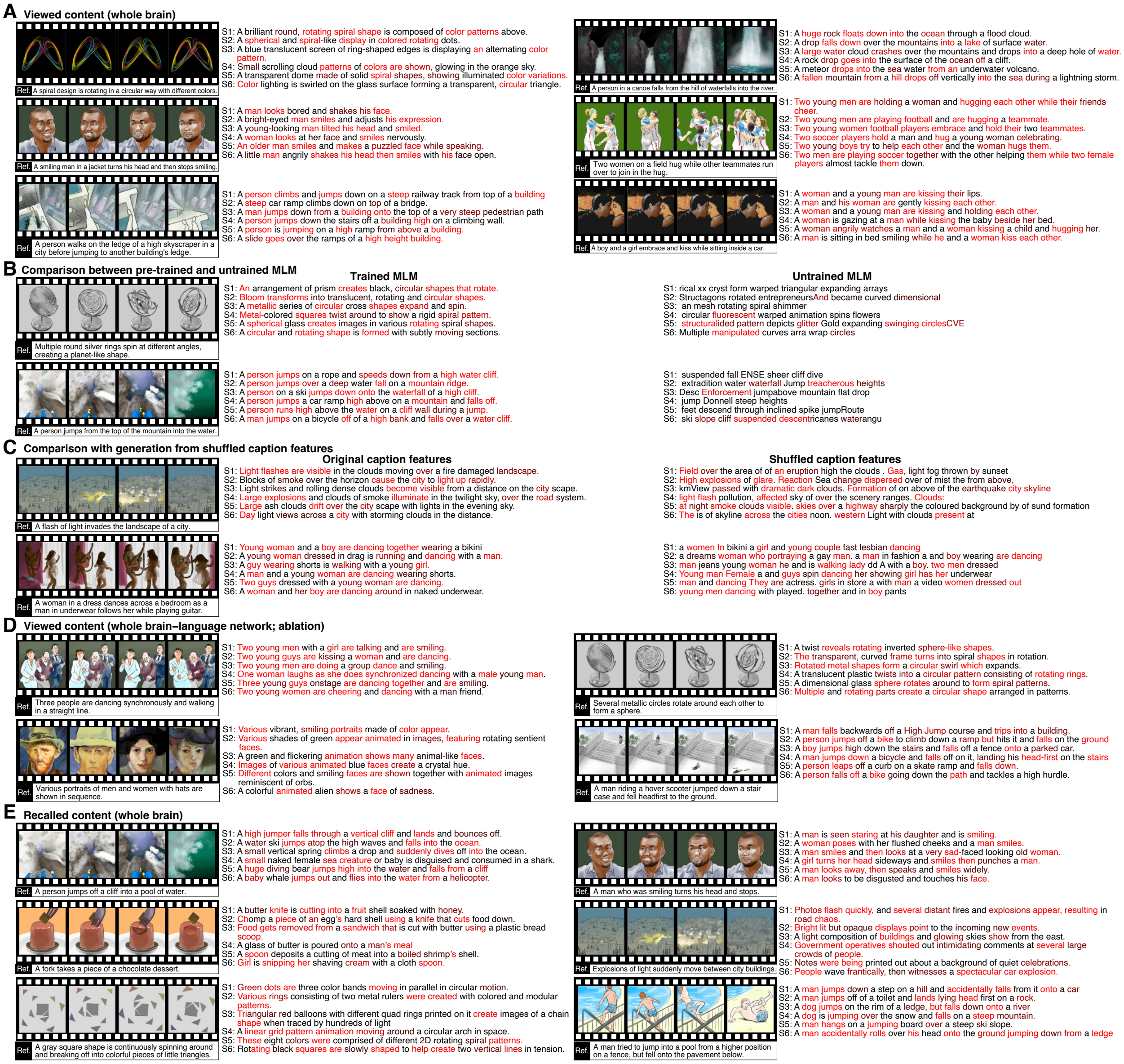

Fig. S3. Examples of generated descriptions.
(**A**) Descriptions of viewed content generated from whole-brain activity. (**B**) Descriptions of viewed content generated with the support of either the pre-trained or untrained MLM model. (**C**) Descriptions of viewed content generated with features predicted by decoders trained based on original captions or those based on captions whose word order was randomly shuffled within each caption. Notably, the descriptions generated for the shuffled condition contained words representing individual components in viewed videos, indicating that semantic features of shuffled captions still convey the word-level semantic information. However, these descriptions lacked the coherence to accurately describe the relations among individual components. (**D**) Descriptions of viewed content generated from the activity of the whole brain except for the language network. (**E**) Descriptions of recalled content generated from whole-brain activity. Conventions are the same as for Fig. 2A.

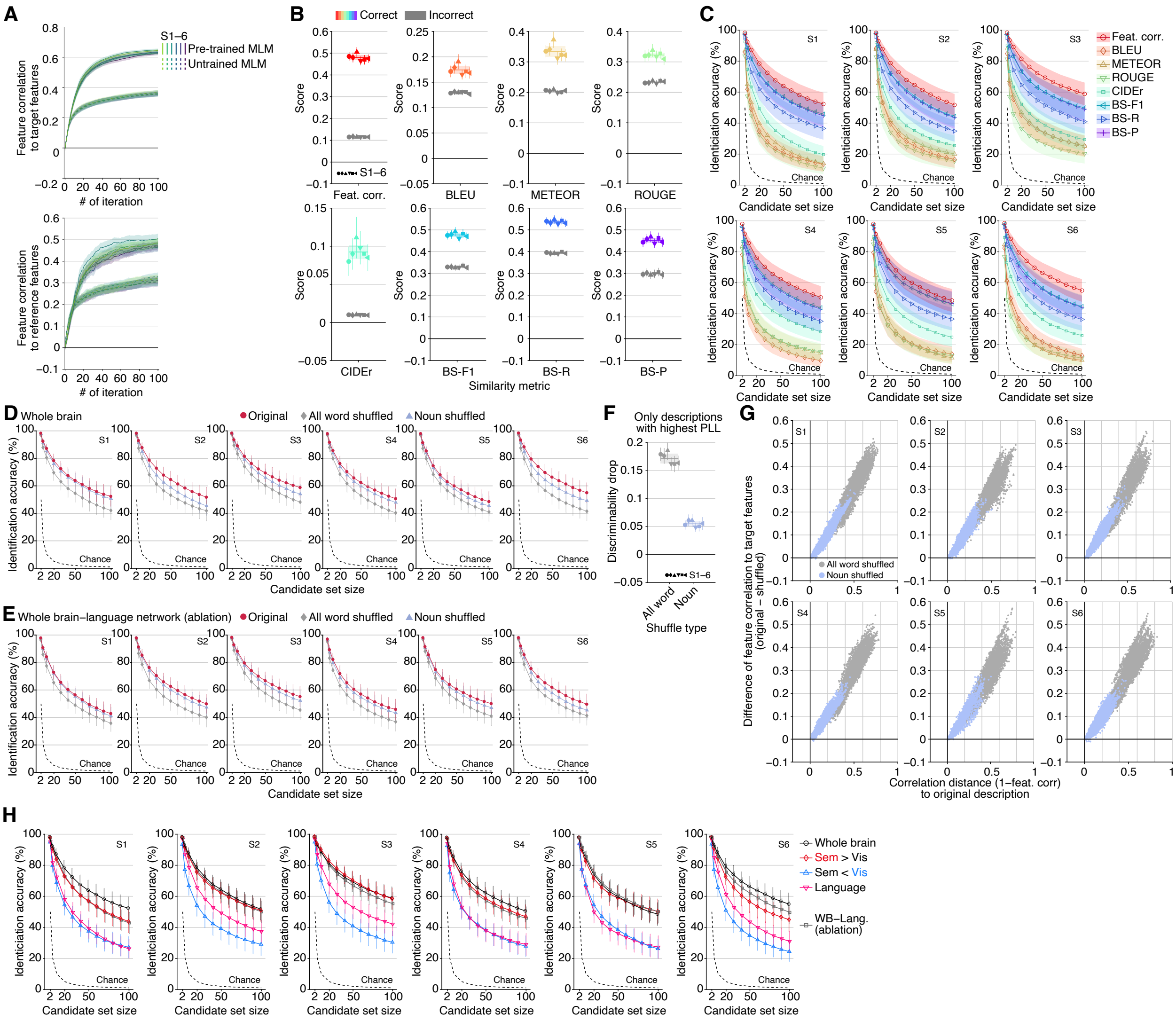

Fig. S4. Text generation performance of viewed content for individual subjects.
(**A**) Feature correlations between semantic features of generated descriptions and those decoded from the brain, as well as those computed from correct references. The generated descriptions supported by the pre-trained MLM model outperformed those supported by the untrained MLM model for all subjects in both similarity measures (Wilcoxon signed-rank test, one-tailed, $P <$ 0.01, FDR corrected across subjects; 100 iterations). (**B**) Raw scores of the similarity between generated descriptions and captions for correct and incorrect references. (**C**) Video identification accuracy. (**D** and **E**) Effects of word-order shuffling on video identification accuracy applied to descriptions generated from whole-brain activity (D) and the activity of the whole brain except the language network (E). (**F**) Effects of word-order shuffling on discriminability using minimally disrupted shuffled descriptions. For each generated description, reductions in discriminability were evaluated by comparing it to the shuffled variants that had the highest pseudo-log-likelihood (PLL) scores, as determined by MLM scoring (*44, 45*). These descriptions were selected from a pool of up to 1,000 shuffled variants created under the all-word or noun-only shuffling conditions. (**G**) Scatterplot of the correlation distances (one minus feature correlation) between the original and shuffled descriptions against the difference in feature

correlations to target features between the original and shuffled descriptions. (**H**) Video identification accuracy obtained from different brain areas. Shades in (A, C), and error bars in (B, D, F, H) indicate 95% C.I. across samples ($N = 72$). Shades in (B, F) indicate 95% C.I. across subjects ($N = 6$).

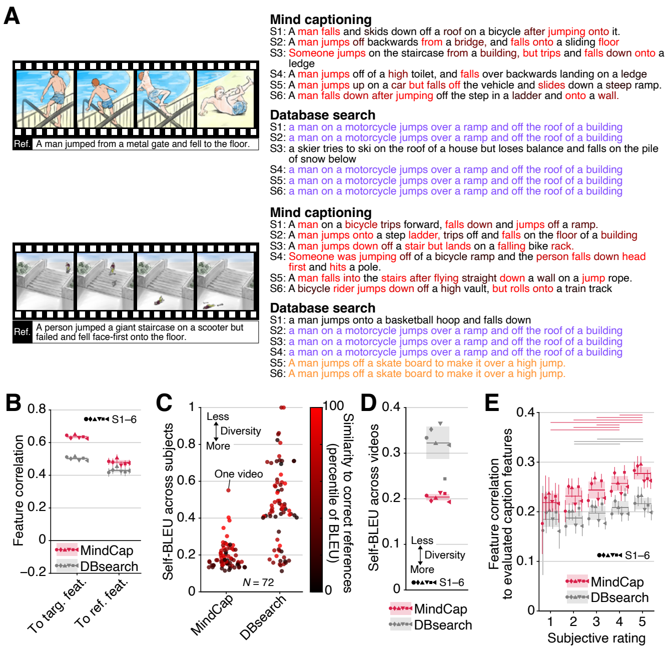

Fig. S5. Comparison with database-search method.
Text generation performance for viewed content was compared between our method and the database (DB) search method (*17*). (**A**) The DB search produced moderately accurate descriptions, but it often produced identical ones for different individuals and videos. (**B**) Our method outperformed DB-search, showing higher feature correlations with both target and reference features (Wilcoxon signed-rank test, one-tailed, $P < 0.01$, FDR corrected across subjects). (**C** and **D**) Diversity (self-BLEU) across subjects (C) and videos (D). Our method exhibited greater diversity than the DB search both across subjects and videos (Wilcoxon signed-rank test, one-tailed, $P < 0.05$). (**E**) Feature correlations at each subjective rating. Both methods showed higher scores for captions with higher ratings (lines above, $P < 0.01$, FDR corrected across pairs, six subjects pooled). Our method showed a higher correlation between ratings and feature correlations than the DB search (mean $r = 0.184$ and 0.134 for mind captioning and DB search, respectively; six subjects averaged; $P < 0.01$, six subjects pooled), demonstrating higher flexibility in generating descriptions better aligned with subjective perceptions (ANOVA, interaction between methods and ratings, $P < 0.01$; six subjects pooled). Shades and error bars indicate 95% C.I. across subjects and samples ($N = 6$ and 72, respectively).

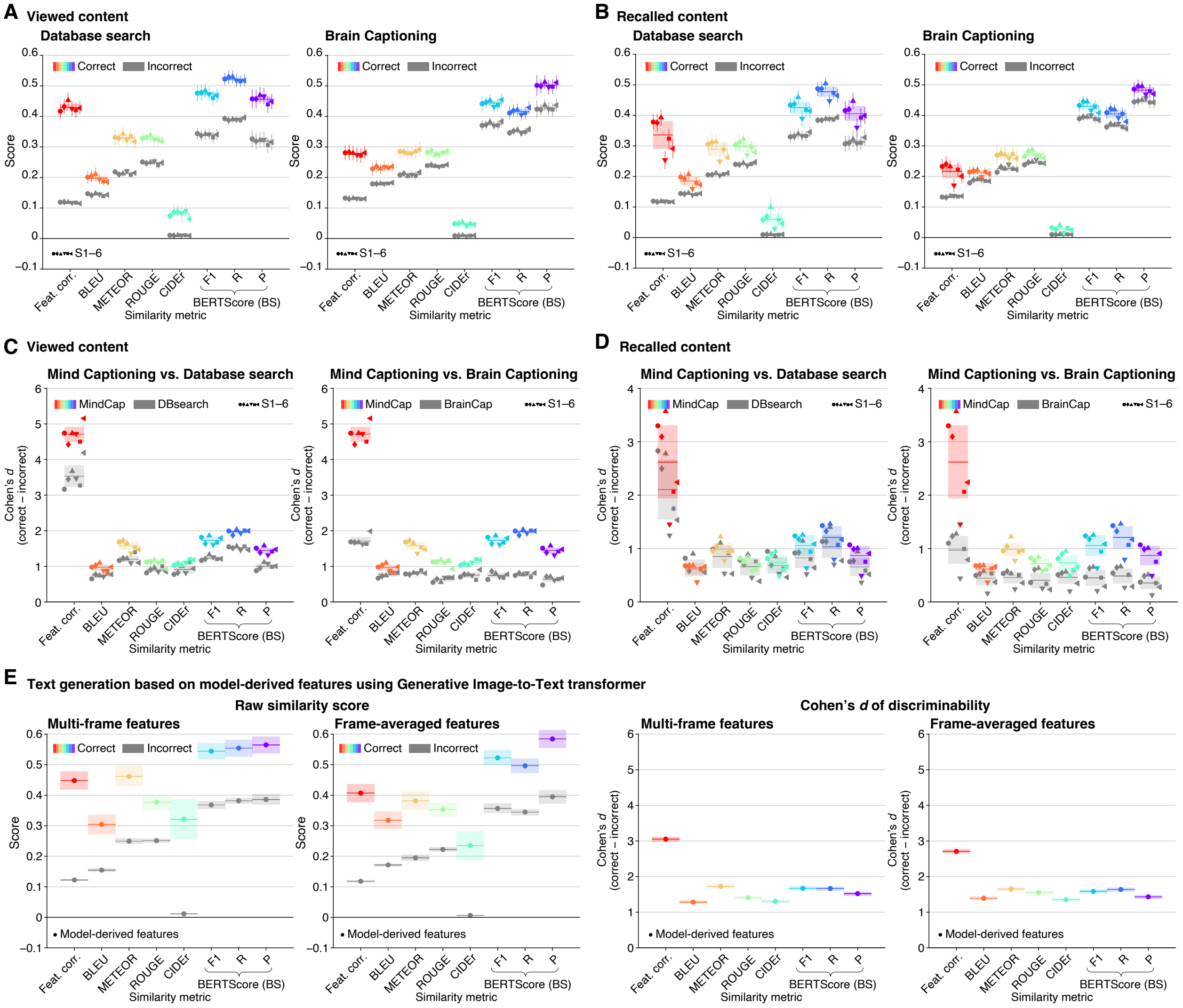

Fig. S6. Performance comparison of brain-to-text decoding methods on video data. We compared the performance of three brain-to-text decoding methods—mind captioning, the database (DB) search (*17*), and brain captioning (*18*)—using our video fMRI data. Brain captioning is a decoding approach that employs a non-linear image captioning model, the *Generative Image-to-text Transformer* (GIT) (*57*) (see Materials and Methods). (**A** and **B**) Raw similarity scores between generated descriptions and captions of correct and incorrect references for viewed (A) and recalled (B) content. (**C** and **D**) Comparisons of Cohen's *d* of discriminability between methods for viewed (C) and recalled (D) content. Overall, mind captioning achieved the highest discriminability in both viewed and recalled conditions, suggesting that it generates descriptions that are more distinguishable from those of irrelevant videos. While brain captioning and DB-search achieved higher raw scores on certain precision-oriented metrics (e.g., BLEU; see also Fig. S4B), this may be due to their tendency to produce descriptions with less lexical and syntactic variability, which surface-level similarity metrics may favor. In contrast, mind captioning generated more semantically discriminative descriptions, even with lower surface similarity. (**E**) Raw similarity scores and Cohen's *d* of discriminability of descriptions generated by GIT using model-derived (not brain-decoded) features. While GIT supports both image and

video captioning—processing either single images or multiple frames—brain captioning is designed for single-image inputs. Therefore, we compared two input configurations for GIT: (1) multi-frame features from uniformly sampled frames and (2) frame-averaged features, obtained by averaging features across all video frames. Although the latter is suboptimal for GIT, its performance was comparable, supporting the validity of using frame-averaged features in brain captioning. For (A) and (B), and (C) and (D), conventions follow those in Fig. 2D and fig. S4B, respectively. Shades in (E) indicate 95% C.I. across samples ($N = 72$).

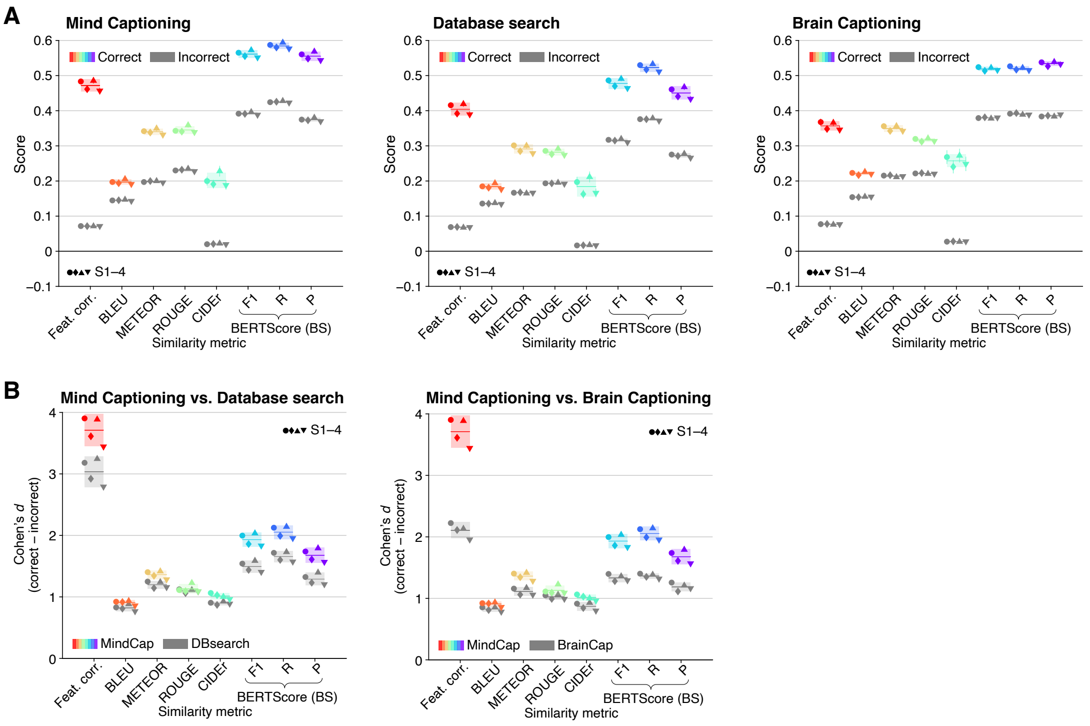

Fig. S7. Performance comparison of brain-to-text decoding methods on NSD image data. Both the database (DB) search (*17*) and brain captioning (*18*) were originally introduced for decoding image-based brain activity; however, in fig. S6, we evaluated them on video-induced data. To complement that comparison, we further evaluated the performance of all three methods, including mind captioning, using image-induced fMRI data from four subjects in the *Natural Scenes Dataset* (NSD) (*39*) (see Materials and Methods). (**A**) Raw similarity scores between generated descriptions and captions of correct and incorrect references for viewed content. (**B**) Cohen's *d* of discriminability between methods for viewed content. Mind captioning achieved the highest discriminability among these methods, demonstrating strong performance even for captioning image-induced brain activity. These results emphasize the superiority of our method and support its robustness across stimulus modalities. For (A) and (B), conventions follow those in Fig. 2D and fig. S4B, respectively.

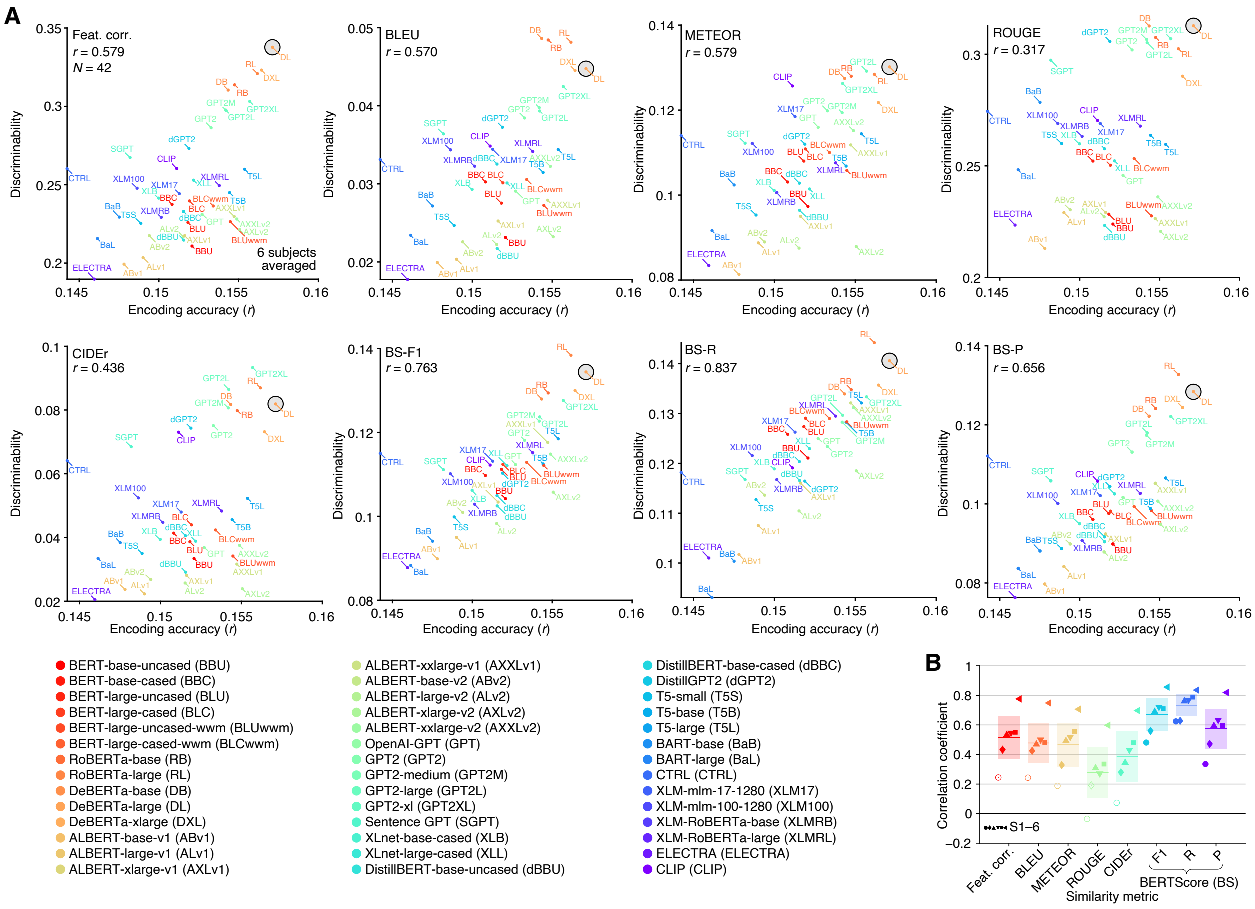

Fig. S8. Relations between encoding accuracy and text generation performance of multiple LMs.

We performed decoding and text generation analyses using features from 42 LMs. We consistently used the DeBERTa-large model for feature correlation evaluation and the RoBERTa-large model to support text generation, respectively. Encoding analysis was performed by training models on the training perception data and testing them on the test perception data. (**A**) Scatterplot of encoding accuracy against discriminability ($N = 42$; dark circle, the model used in the main analysis). For each model, discriminability scores were averaged across samples, and encoding accuracy was averaged across voxels in the whole brain. The discriminability scores were positive for all models, indicating the robustness of our method to the choice of LMs. (**B**) Correlation coefficients between discriminability and encoding accuracy for all models. We found significantly positive correlations for most metrics and subjects, except S1 (filled markers, t-test, one-tailed, $P < 0.01$, FDR corrected across metrics and subjects), suggesting that LMs with features more aligned with brain representations could help generate descriptions that accurately capture the viewed video content. Shades and error bars indicate 95% C.I. across samples ($N = 72$) and subjects ($N = 6$), respectively.

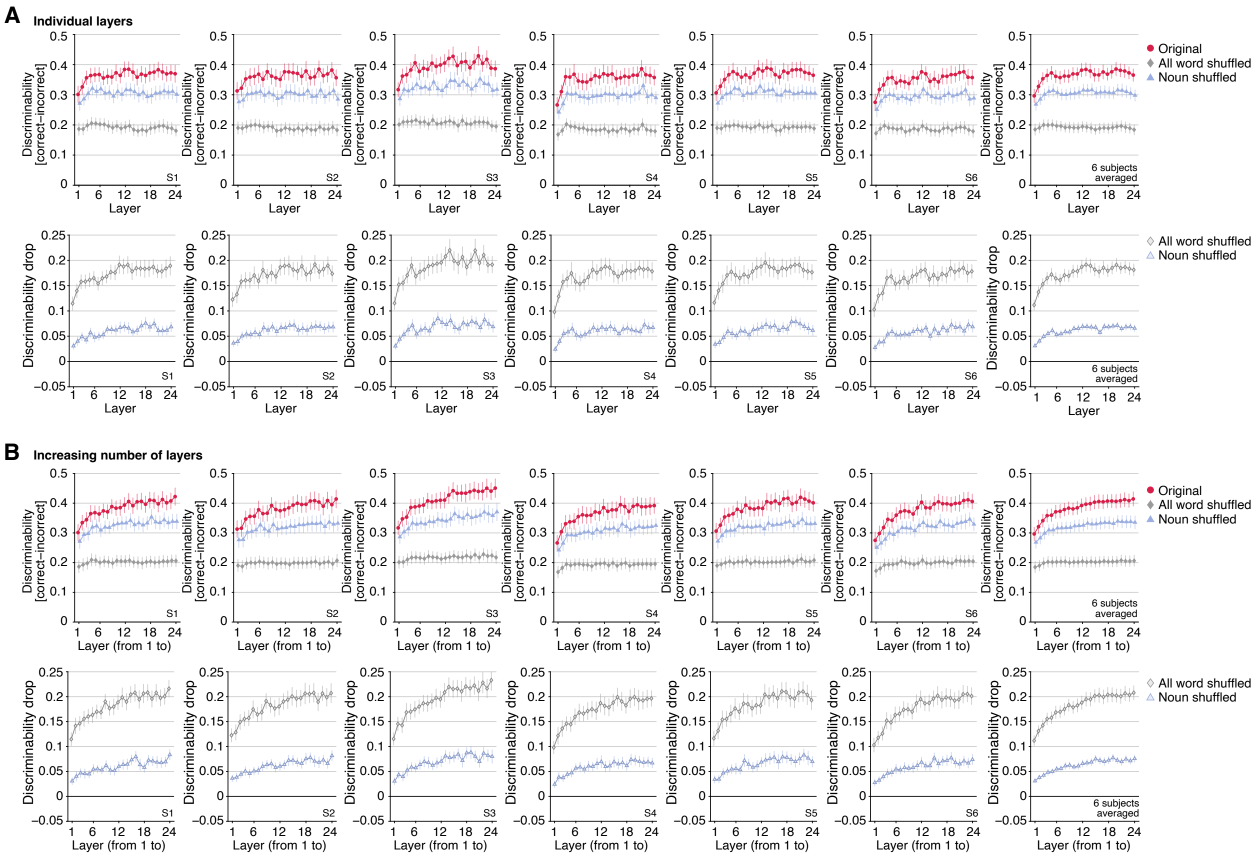

Fig. S9. Effects of shuffling on discriminability with varying numbers of layers.
The text generation analysis was conducted using decoded features with varying numbers of LM layers (24 layers from DeBERTa-large; individual layers or increasing number of layers). We evaluated the impact of shuffling on the discriminability of the descriptions generated with the varying number of layers. To compare the results from different layer conditions, we consistently used the feature correlations computed using all layers of the DeBERTa-large model as a fixed metric. (**A** and **B**) Discriminability and the drop in discriminability caused by shuffling for descriptions generated with individual layers (A) and with increasing number of layers (B). Overall, text generation with deeper layers tended to result in higher discriminability (mean r = 0.087 for (A); mean r = 0.189 for (B); t-test, one-tailed, $P < 0.05$, FDR corrected across subjects). Furthermore, the drop in discriminability caused by shuffling became significantly larger when deeper layers were included in the analysis. Error bars indicate 95% C.I. across samples (N = 72; mean r = 0.180 [all] and 0.187 [noun] for (A); mean r = 0.259 [all] and 0.214 [noun] for (B); t-test, one-tailed, $P < 0.05$, FDR corrected across subjects).

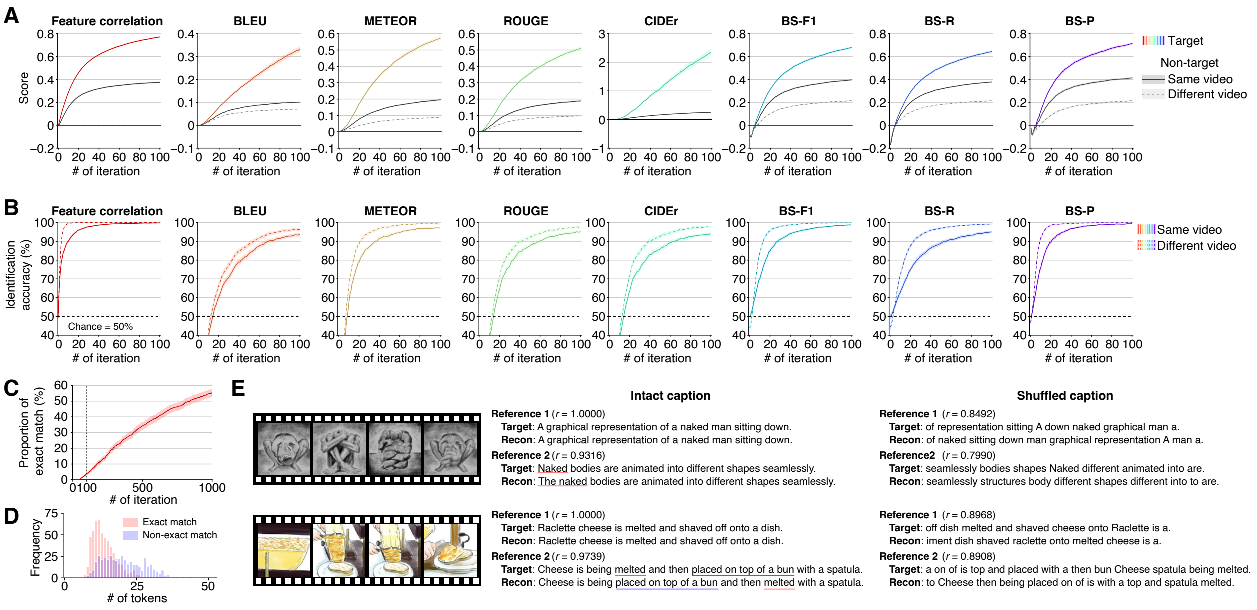

Fig. S10. Performance of caption reconstructions based on features computed from reference captions. To examine how accurately our text optimization method can reconstruct original descriptions from semantic features, we performed a text generation analysis using model-derived (not brain-decoded) features from reference captions of 50 randomly selected videos in the training perception data (20 captions per video; $N = 1{,}000$). (**A**) Raw similarity scores between reconstructed descriptions and target captions, along with non-target captions from the same and different videos. (**B**) Pairwise caption identification accuracy based on reconstructed descriptions using candidate captions from the same or different videos. Similarity scores to target captions increased during optimization, with identification accuracy—particularly with feature correlation—approaching 100% after 100 iterations, even for captions from the same video (99.83%), justifying 100 iterations as a computationally efficient and effective setting. (**C**) Proportion of exact matches between reconstructed and original captions across 1,000 iterations. The proportion of exact matches was 4.0% after 100 iterations but increased to 55.4% after 1,000 iterations, indicating that extended optimization can improve reconstruction fidelity. (**D**) Distributions of token counts in target captions. Longer captions were generally harder to reconstruct exactly. (**E**) Examples of reconstructed captions from intact and shuffled references. Exact reconstructions yielded a feature correlation (r) of 1.0, whereas minor word omissions or reordering reduced correlations, demonstrating that the semantic feature space retains sufficient information for accurate reconstruction, even with token-averaged features (see Materials and Methods). However, reconstructions of shuffled captions yielded only 0.2% exact matches even after 1,000 iterations, while recovering most target words and reproducing incoherent structure. These results suggest that while reconstruction difficulty increases with longer or less coherent captions—reflecting optimization limitations and residual MLM priors rather than information loss—our method is highly effective in generating descriptions aligned with target features through iterative feature-guided optimization.

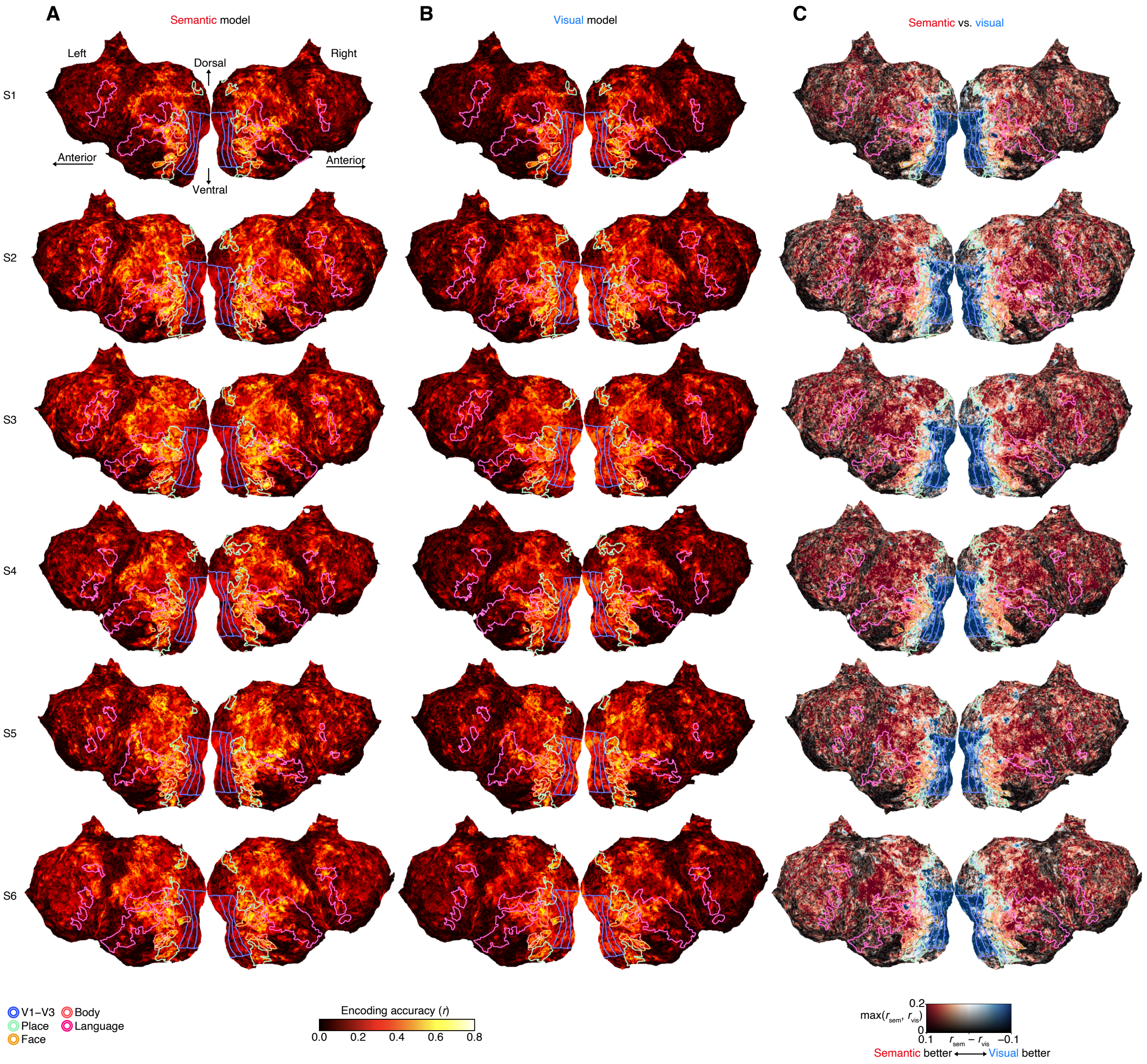

Fig. S11. Voxel-wise encoding model performance.
(**A**) Encoding accuracy of the semantic encoding model. (**B**) Encoding accuracy of the visual encoding model. (**C**) Comparison of encoding accuracy between the semantic encoding model and visual encoding model. Across all subjects, a consistent pattern emerged in which voxels in the occipital visual areas were more accurately predicted by the visual model, whereas voxels in more anterior regions were better predicted by the semantic model. The shift in model superiority occurs at the midpoint of the category-selective regions, between their posterior and anterior halves.

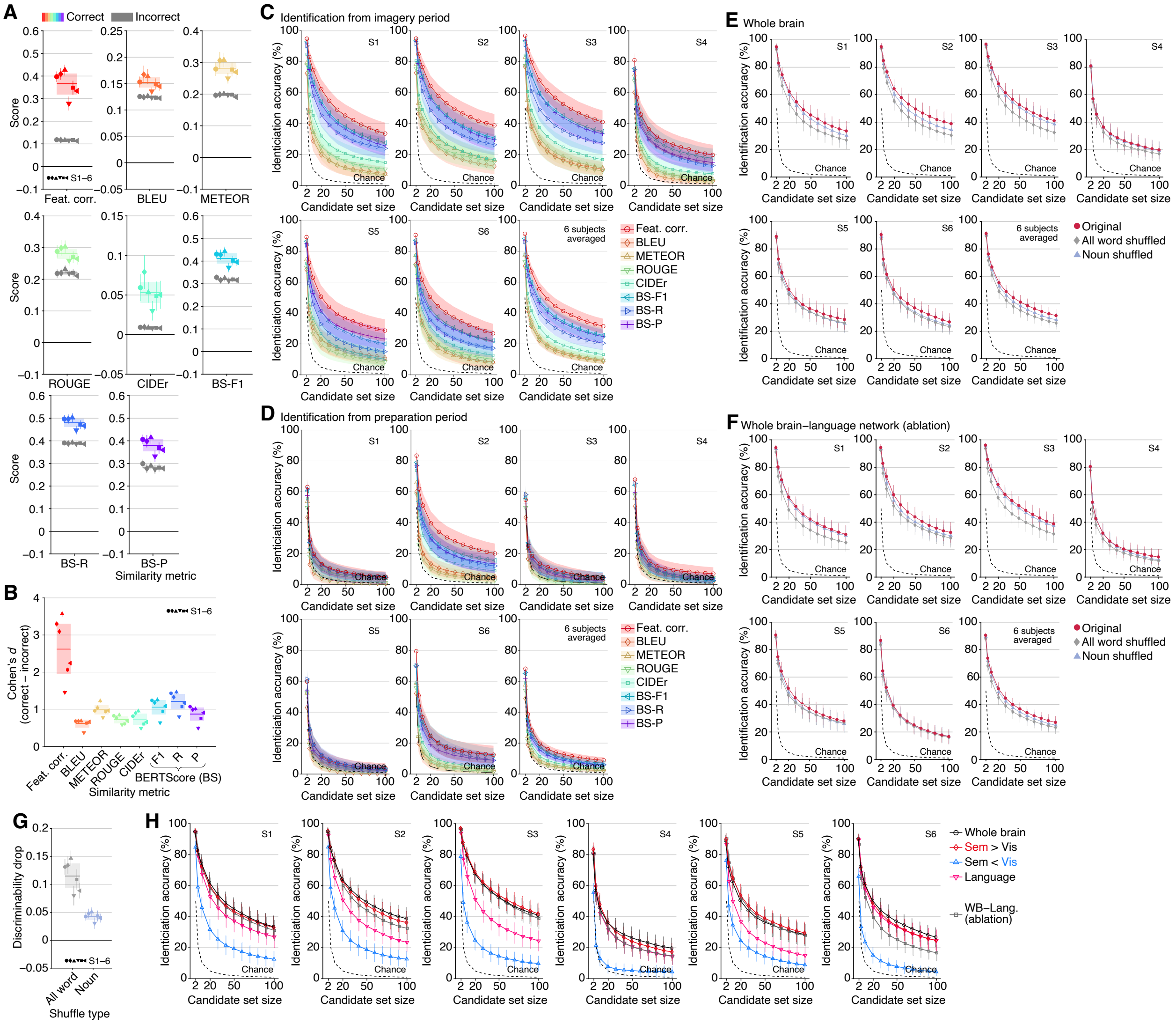

Fig. S12. Text generation performance of recalled content for individual subjects. (**A**) Raw scores for the similarity between generated descriptions and captions for correct and incorrect references based on multiple similarity metrics. (**B**) Cohen's d of the discriminability. The generated descriptions showed significantly high discriminability across all metrics and subjects (Wilcoxon signed-rank test, one-tailed, $P < 0.01$, FDR corrected across metrics and subjects). (**C** and **D**) Identification accuracy of recalled videos from brain activity during the imagery period (C) and the preparation period (D). (**E** and **F**), Effects of word-order shuffling on identification accuracy of recalled videos applied to descriptions generated from whole-brain activity (E) and the activity of the whole brain except the language network (F). (**G**) Effects of word-order shuffling on discriminability. The word-order shuffling resulted in significant drops in discriminability even without using the language network (Wilcoxon signed-rank test, one-tailed, $P < 0.01$, FDR corrected across subjects). (**H**) Identification accuracy of recalled videos from different brain areas. Shades in (C, D) and error bars in (A, E–H) indicate 95% C.I. across samples ($N = 72$). Shades in (A, B, G) indicate 95% C.I. across subjects ($N = 6$).

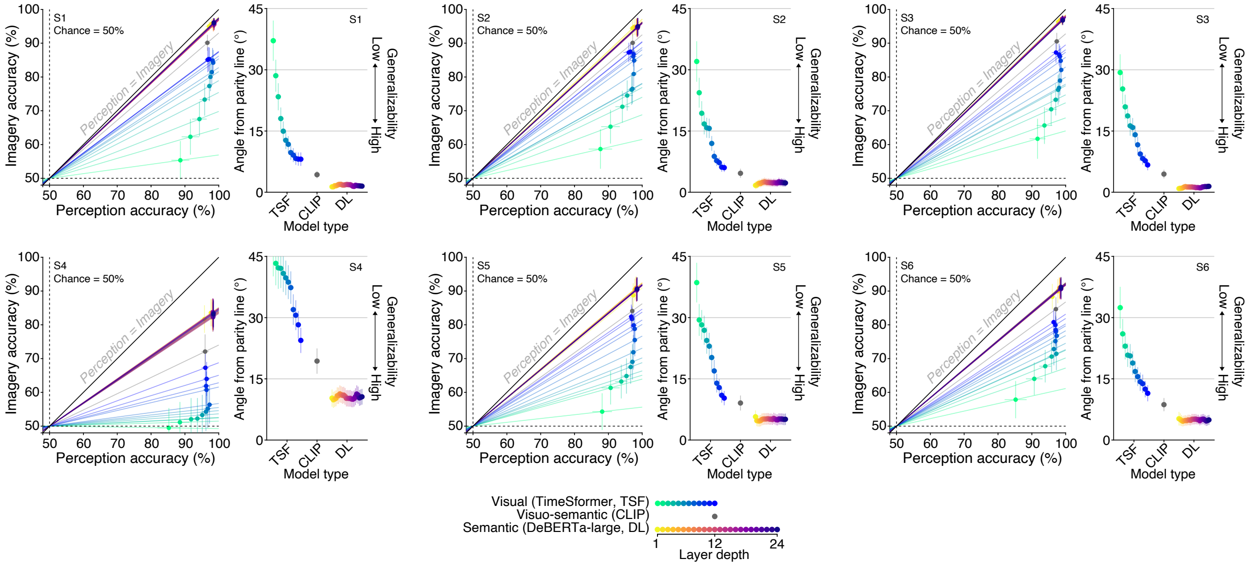

Fig. S13. Comparison of feature-based pairwise video identification accuracy between perception and imagery for individual subjects.

Across all subjects, generalizability—defined as the angle between the parity line and the line connecting chance level (50%) with the observed accuracies—was consistently highest for semantic features across all layers. These results provide robust evidence that semantic features enable perception-trained decoders to effectively generalize for decoding mental content, even at the single-subject level. Conventions are the same as for Fig. 4E.

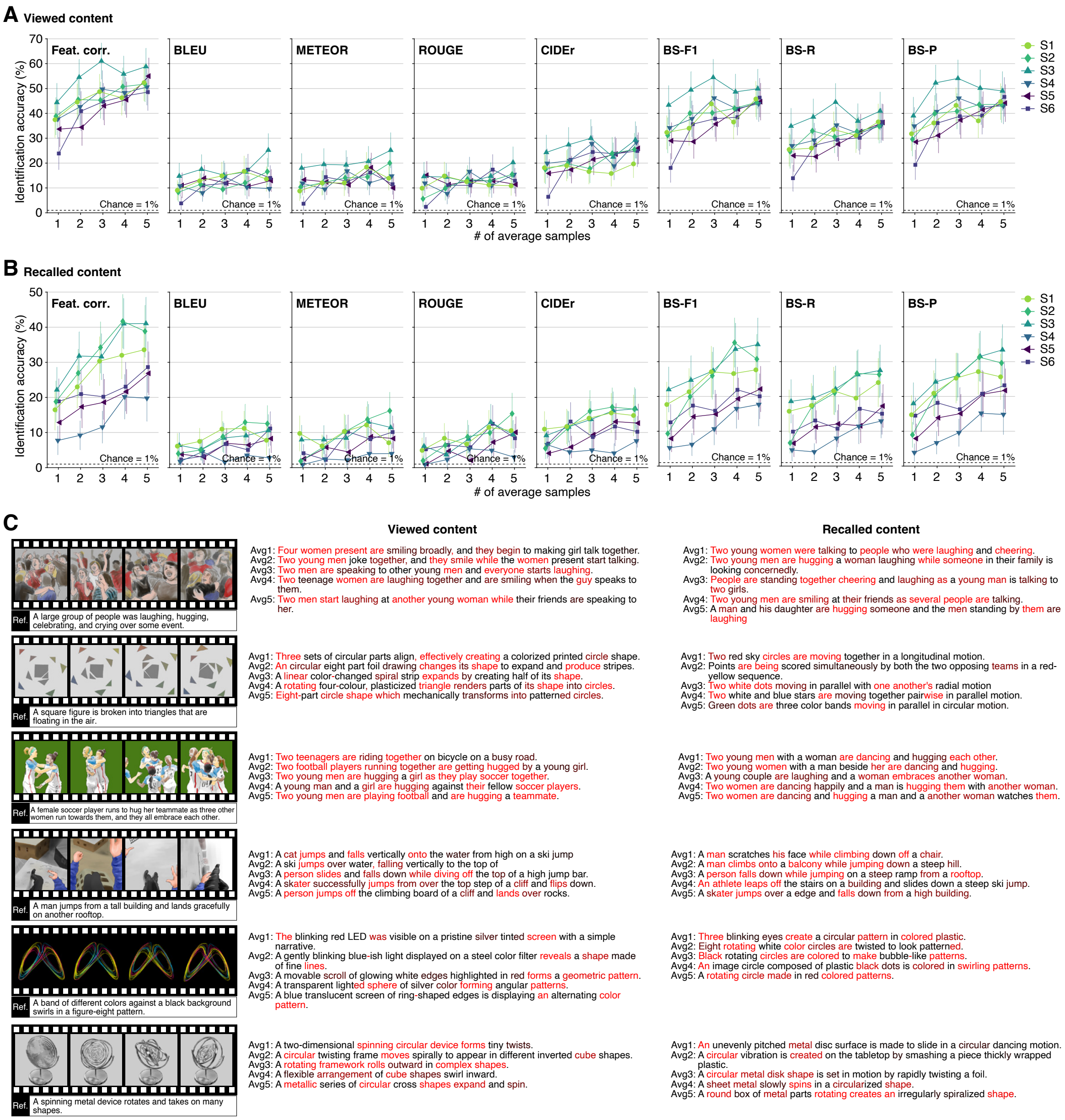

Fig. S14. Performance of text generation with different numbers of averaged samples. The feature decoding and text generation analyses were performed with brain activity averaged across different numbers of samples. (**A** and **B**) Video identification accuracy of viewed (A) and recalled (B) content with varying numbers of averaged samples. The video identification analysis was performed with 100 candidates (chance = 1%; error bars, 95% CI across samples). (**C**) Descriptions generated using different numbers of averaged samples. For each video, generated descriptions from the same subjects are shown for both viewed and recalled content. The analysis revealed that the quality of the generated descriptions improved as the number of averaged samples increased, indicating that the performance was limited by noise in the fMRI signal of the test data. Meanwhile, the descriptions generated from fMRI activity in single trials reasonably captured the content of both viewed and recalled videos. These results suggest that our method is capable of generating moderately accurate descriptions of both viewed and recalled content, even when using single-trial fMRI activity.
